# CEACAM7 Promotes Tumor Progression by Regulating GCNT3 Expression in Lung Adenocarcinoma

**DOI:** 10.3390/cancers18213486

**Published:** 2026-10-29

**Authors:** Qing Zhang, Qi-Liang Feng, Rong-Rong Chen, Ni-Ni Wang, Cheng-Shi Zhang, Yun-Feng Zhao

**Affiliations:** 1 Department of Respiratory Disease, Punan Hospital, Pudong New Area, Shanghai 200125, China; 2 Department of Respiratory Disease, Punan Branch of Renji Hospital, Shanghai Jiaotong University School of Medicine, Shanghai 200125, China

**Keywords:** LUAD, CEACAM7, GCNT3, migration, proliferation, invasion

## Abstract

**Background**: Lung adenocarcinoma (LUAD) is a global epidemic with extremely high lethality, yet its exact molecular mechanisms remain under-explored. Therefore, the study of molecular regulators of the developmental process of LUAD is of crucial importance for developing innovative therapeutic approaches to enhance the survival rate of individuals with LUAD. **Methods**: Through bioinformatics analyses of several public gene databases, we thoroughly examined the expression, clinical significance, co-expressed genes, and signal transduction pathways of CEACAM7 in LUAD. Reverse transcription quantitative PCR (RT-qPCR) and Western blotting (WB) were utilized to detect the CEACAM7 expression in clinical LUAD tissues. The CCK-8 method, WB, colony formation, Transwell, wound healing, and immunoprecipitation assays were utilized to explore the molecular mechanism of CEACAM7′s role in LUAD. The tumorigenic role of CEACAM7 was further validated in vivo in a subcutaneous xenograft model established in female BALB/c nude mice (*n* = 5 per group). **Results**: CEACAM7 is significantly overexpressed in LUAD, and its upregulation is linked to tumor aggressiveness and poor prognosis. Our experimental results suggest that regulation of CEACAM7 expression, either through up- or down-regulation, results in enhancement or suppression of growth and invasion of H1650 cells, respectively. In H1650 cells, CEACAM7 was shown to directly interact with GCNT3 and trigger growth and invasion of H1650 cells and epithelial–mesenchymal transition (EMT). In vivo, knockdown of CEACAM7 significantly suppressed xenograft tumor growth in nude mice. **Conclusions**: Our findings demonstrating the biological function of CEACAM7 in LUAD both in vitro and in vivo provide new insights and underline its capability as a predictive biomarker and therapeutic target.

## Simple Summary

Lung adenocarcinoma is the most common type of lung cancer and remains difficult to treat once it has spread. In this study, we examined CEACAM7, a protein belonging to a family of molecules that help cells stick together and communicate. We found that CEACAM7 is far more abundant in lung adenocarcinoma tissue than in normal lung tissue, and that high levels are associated with more aggressive tumors. Using lung cancer cells and a mouse tumor model, we showed that reducing CEACAM7 slows tumor growth and makes the cells less able to migrate and invade, whereas increasing it has the opposite effect. CEACAM7 appears to act by raising the levels of another protein, GCNT3, which modifies sugars on the cell surface. These findings suggest that CEACAM7 could serve as a biomarker and a potential target for new treatments.

## 1. Introduction

Lung cancer (LC) is the major cause of cancer morbidity and mortality in men [1]. Lung adenocarcinoma (LUAD) is the most predominant type of non-small cell LC (NSCLC), characterised by a dense lymphocytic infiltrate and a tendency to metastasise at an early stage [2]. Recently, the prognosis for individuals with LUAD has improved dramatically as the treatment of LUAD has gradually evolved from traditional radical lobectomy or sublobar resection to personalised treatment tailored to the genetic profile of the cancer cells [3,4]. Despite the tremendous development of LUAD screening technology, many individuals are diagnosed in the middle or late stages owing to the lack of obvious symptoms in the early stages. LUAD is still prone to recurrence and death, and targeted therapies for LUAD, as well as immunotherapy and other treatments, continue to make progress, but they all suffer from obvious shortcomings; patients inevitably develop drug resistance, and their overall survival rate at 5 years is still less than 15% [5,6]. Thus, it is important to examine the mechanisms of LUAD occurrence and metastasis, which may yield novel approaches for LUAD treatment.

Carcinoembryonic antigen (CEA)-related cell adhesion molecules (CEACAMs), parts of the immunoglobulin (Ig) superfamily, exhibit diverse biological functions, ranging from cell adhesion and tumor progression to signal transduction and phagocytosis. Specific family members have been characterized in distinct physiological and pathological contexts; for instance, CEACAM3 mediates the phagocytosis of pathogens in granulocytes via immunoreceptor tyrosine-based activation motif (ITAM)-like sequences, while CEACAM1 regulates insulin clearance and immune responses [7,8]. In contrast, CEACAM1, 5, and 6 are widely known to be overexpressed in NSCLC. In the specific context of NSCLC, several members act as key drivers of malignancy through defined mechanisms. CEACAM1 overexpression has been directly correlated with increased angiogenic potential in NSCLC tissues, facilitated by stimulation of the NF-κB signaling pathway [9,10]. CEACAM5 facilitates tumor progression by stimulating cell growth and migration in vitro and in vivo, mechanisms that have been linked to the regulation of the p38-Smad2/3 pathway [11]. Notably, CEACAM6 is significantly upregulated in LUAD, particularly in association with epidermal growth factor receptor (EGFR) mutations; it modulates the p-ERK1/2 signaling pathway to inhibit apoptosis, and its involvement in EMT has been linked to resistance against EGFR-tyrosine kinase inhibitors (TKIs) [12].

In contrast to these extensively characterized members, CEACAM7 exhibits distinct structural features. Unlike the complex, multi-domain structure of CEACAM5, CEACAM7 is compact, typically containing only one N-terminal IgV-like domain and one or two IgC2-like domains [13]. Regarding signal transduction, autonomous signaling is primarily restricted to transmembrane family members possessing functional cytoplasmic domains. For instance, CEACAM1 regulates inhibitory signaling via Immunoreceptor Tyrosine-based Inhibitory Motifs (ITIMs), whereas CEACAM3 mediates phagocytic signaling through ITAM-like sequences [14]. Furthermore, unlike the transmembrane protein CEACAM1, which possesses a cytoplasmic tail capable of intracellular signaling (e.g., via ITIMs), CEACAM7 is anchored to the membrane through a glycosylphosphatidylinositol (GPI) moiety and possesses neither a transmembrane domain nor a cytoplasmic tail [15,16]. This structural uniqueness implies that CEACAM7 likely functions through lateral interactions with other membrane proteins rather than autonomous signal transduction. Consequently, the mechanisms established for CEACAM1, CEACAM5 and CEACAM6 in LUAD, all of which depend on their transmembrane and cytoplasmic domains, cannot be assumed to apply to CEACAM7. Whether CEACAM7 contributes to LUAD at all, and by what means, has therefore remained an open question. Historically, CEACAM7 expression was restricted to the colon and pancreas, leading to its oversight in LC research [17,18]. However, our preliminary bioinformatics analysis revealed that CEACAM7 is significantly upregulated in LUAD, hinting at a unique pathogenic role. Given this unexpected expression pattern and the scarcity of studies linking CEACAM7 to respiratory malignancies, we investigated whether CEACAM7 functions as an oncogene in LUAD and explored its underlying mechanisms.

## 2. Materials and Methods

### 2.1. Bioinformatic Analysis

A bioinformatics investigation was conducted using The Cancer Genome Atlas (TCGA) (https://portal.gdc.cancer.gov/, accessed on 10 March 2024). RNA-seq gene expression data and clinical records were acquired for analysis. Using the TCGA database, we investigated the levels of CEACAM7 and GCNT3 in 58 non-cancerous tissues and 538 LUAD tissues. To validate the CEACAM7 expression in an independent cohort, we utilized the Gene Expression Omnibus (GEO) database (https://www.ncbi.nlm.nih.gov/geo/, accessed on 10 March 2024). The dataset GSE118370 was selected for validation analysis. We conducted differential expression analysis via the limma (version 3.52.4) R package. For between-group comparisons of gene expression, the Wilcoxon rank-sum test was applied for unpaired samples (tumor vs. normal), while the Wilcoxon signed-rank test was used for paired samples (tumor vs. matched adjacent normal). Additionally, diagnostic receiver operating characteristic (ROC) curves were constructed via the pROC (version 1.18.0) package to evaluate diagnostic value. Correlation measures were evaluated using the Pearson method through the cor.test function in R. Relevant genes exhibiting the most significant positive correlation coefficients with CEACAM7 were selected. Differentially expressed genes (DEGs) were defined by |logFC| > 1 and an adjusted *p* value < 0.05, with *p* values adjusted for multiple testing using the Benjamini–Hochberg method. The correlation between CEACAM7 and GCNT3 expression was assessed by Pearson correlation analysis.

### 2.2. Clinical Tissues

We collected 27 pairs of LUAD tissues and matched adjacent normal tissues from patients who underwent surgical resection at Punan Hospital, Pudong New Area, Shanghai, from March 2023 to March 2024. Patients were enrolled consecutively and without selection on the basis of sex, so the cohort comprised both male and female patients. Data were accessed for research purposes on 15 April 2024. To confirm the validity of the controls, normal tissues were obtained from areas distal to the tumor margin. The study was conducted in accordance with the Declaration of Helsinki and was approved by the Ethics Committee of Punan Hospital, Pudong New Area, Shanghai (Approval No. 2023-EC-0456). Written informed consent was obtained from each participant prior to enrolment. Upon collection, tissue samples were divided into two parts: one was paraformaldehyde-fixed for histological analysis, and the other was immediately snap-frozen in liquid nitrogen and stored at −80 °C for RNA and protein isolation. All diagnoses were confirmed independently by two pathologists in a blinded manner.

### 2.3. Cell Lines and Culture

For our in vitro studies, we acquired the BEAS-2B human normal lung epithelial cell line, along with the A549, PC-9, and H1650 human NSCLC cell lines, from the Cell Bank/Stem Cell Bank, Chinese Academy of Sciences (Shanghai, China). Human embryonic kidney 293T (HEK293T) cells were purchased from Procell Life Science & Technology Co., Ltd. (Wuhan, China). All cell lines were supplied with short tandem repeat (STR) authentication certificates from the suppliers, and the reported profiles matched the reference profiles deposited in Cellosaurus; all cell lots were certified mycoplasma-free. These cells were grown in DMEM medium (Gibco, Grand Island, NY, USA) with 1% penicillin/streptomycin and 10% fetal bovine serum, and incubated at 37 °C in a humidified environment with 5% CO_2_.

### 2.4. Cell Transfection

To construct overexpression models, full-length cDNA sequences of CEACAM7 and GCNT3 were cloned into the GV320 vector (GeneChem, Shanghai, China). The empty GV320 vector was considered the negative control (Vector). For gene suppression, the synthesis of short hairpin RNAs (shRNAs) targeting CEACAM7 and GCNT3, as well as a scrambled non-targeting negative control (shNC), was conducted by GeneChem. These are the target sequences: sh-NC (5′-TTCTCCGAACGTGTCACGT-3′), sh-CEACAM7 (5′-ATCTTACCAAATTAAAGAGGC-3′), and sh-GCNT3 (5′-AACGTAGGTATTCTTCTAAGC-3′). The transfection procedure was conducted as per the manufacturer’s guidelines. We established stable cell lines by puromycin selection, and transfection efficacy was verified by RT-qPCR and Western blotting (WB).

### 2.5. RNA Isolation and RT-qPCR

Total RNA isolation was conducted as per the manufacturer’s guidelines using TRIzol reagent. Then, the extracted RNA reverse transcription was conducted to cDNA via the PrimeScript reverse transcription kit from TaKaRa (Kusatsu, Japan). Subsequently, we conducted qPCR on an ABI 7900 Real-Time PCR System (Applied Biosystems, Foster City, CA, USA) utilizing Applied Biosystems’ SYBR Green kit. The target gene levels were detected via the 2^−∆∆CT^ technique, with GAPDH acting as the internal reference gene. These are the primer sequences used Table 1:

### 2.6. Western Blot

Total protein isolation from cells and lung tissues was conducted via RIPA lysis buffer with protease inhibitors (Bimake, Houston, TX, USA). Protein level was assessed via a BCA protein assay kit (Beyotime, Shanghai, China). After separating equal protein quantities by 8–10% SDS-PAGE, we transferred them onto PVDF membranes. Subsequent to a 1-h blockage with 5% skimmed milk at room temperature, the incubation of membranes was conducted overnight at 4 °C with these primary antibodies: anti-CEACAM7 (DF9346, Affinity Biosciences, Cincinnati, OH, USA), anti-GCNT3 (ab77728, Abcam, Cambridge, UK), anti-E-cadherin (20874-1-AP, Proteintech, Rosemont, IL, USA), anti-Vimentin (5741, Cell Signaling Technology, Danvers, MA, USA), anti-Slug (AF4002, Affinity Biosciences), anti-N-cadherin (13116, Cell Signaling Technology), anti-ZEB1 (DF7414, Affinity Biosciences), and anti-GAPDH (AB-P-R 001, Hangzhou Goodhere Biotechnology, Hangzhou, China). Subsequently, an incubation of the membranes was conducted with horseradish peroxidase (HRP)-conjugated secondary antibodies. We observed protein bands via enhanced chemiluminescence (ECL) (Bio-Rad Laboratories, Hercules, CA, USA). Semi-quantification of protein levels was conducted via ImageJ (version 1.53t), with GAPDH acting as the loading control. The original, uncropped Western blot images are provided as File S1.

### 2.7. Cell Counting Kit 8 (CCK-8) Assay

Cell proliferative activity was estimated via the CCK-8 assay. About 5 × 10^3^ cells were grown in 96-well plates with 100 μL of medium per well, and incubation was conducted for 0, 24, 48, and 72 h in a 5% CO_2_, 37 °C environment. Following this, we treated each well with 10 μg of CCK-8 solution and incubated for 2 h away from light. The absorbance at 450 nm was read on a microplate reader (BioTek, Winooski, VT, USA).

### 2.8. Colony Formation Assay

We inoculated groups of cells in 6-well plates (1000 cells/well). They were cultivated in 5% CO_2_ at 37 °C, and the medium was replaced every two days to maintain the cell growth conditions. Cell colony staining was performed after 14 days by fixing the cells with 4% paraformaldehyde for 15 min and then staining with 0.1% crystal violet for 15 min.

### 2.9. Cell Scratch Test

To exclude any contribution of cell proliferation, cells were pretreated with mitomycin C (10 μg/mL) for 2 h at 37 °C, washed three times with PBS and then used in the scratch assay. H1650 cells were cultivated in 6-well plates, and upon reaching 90% fusion, the cells were scraped with the tip of a 200 μL sterile plastic pipette, and then PBS rinsing was conducted to remove the shed cells, and the cells were cultivated for 24 h. The cells were observed and photographed via an inverted light microscope (Olympus, Tokyo, Japan) at 0 and 24 h to measure the width of the scratches of the cells of the several groups at different times, and the ability of the cells to heal scratches was measured by comparing the ratios between the widths of the cells’ scratches at the different time points and the widths of the initial scratches.

### 2.10. Transwell Assay

The invasive capacity of H1650 cells was analyzed with 24-well polycarbonate films and substrates. Cells were inoculated in serum-free DMEM medium in the top chamber, and DMEM medium with 10% fetal bovine serum was applied to the bottom chamber. An incubation was conducted for 24 h. Cells passing through membranes underwent fixation with 4% paraformaldehyde and staining with 0.1% violet crystals for 3 min, and cells in the bottom chamber were photographed and counted.

### 2.11. Immunoprecipitation (Co-IP) Assay

To validate the physical interaction between CEACAM7 and GCNT3, HEK293T cells were selected as the experimental model because of their increased transfection efficiency and capacity for robust protein expression. We harvested and lysed the cells in ice-cold IP lysis buffer with protease and phosphatase inhibitors. The lysates were spun to collect protein-rich supernatants. Subsequently, the incubation of supernatants was conducted overnight at 4 °C with specific antibodies. For exogenous Co-IP assays involving tagged proteins, mouse anti-Flag (M185-3, MBL, Tokyo, Japan) and rabbit anti-HA (ab9110, Abcam) were utilized. For endogenous interaction validation, rabbit anti-CEACAM7 (DF9346, Affinity Biosciences) and mouse anti-GCNT3 (sc-398646, Santa Cruz Biotechnology, Dallas, TX, USA) were employed. Then, the incubation of immune complexes was conducted with Protein A/G agarose beads. We thoroughly rinsed the beads with lysis buffer to remove non-specific binding, resuspended them in 5× SDS loading buffer, and boiled them. The resulting samples were subjected to SDS-PAGE and WB to analyze protein interactions.

### 2.12. In Vivo Tumorigenesis Assay

Female BALB/c nude mice (four weeks old) were purchased from Shanghai SLAC Laboratory Animal Co., Ltd. (Shanghai, China) and maintained under specific pathogen-free (SPF) conditions with ad libitum access to sterilized food and water. All animal procedures were performed in accordance with the National Guidelines for the Care and Use of Laboratory Animals (Ministry of Science and Technology of China) and the ARRIVE guidelines (https://arriveguidelines.org, accessed on 5 August 2026), and were approved by the Animal Ethics Committee of Yusu Life Sciences (Shanghai) Co., Ltd. (Approval No. YS-m202607007, approved on 28 July 2026). H1650 cells stably expressing sh-NC or sh-CEACAM7 were resuspended in PBS, and 100 μL of cell suspension (2 × 10^6^ cells) was subcutaneously injected into the right flank of each mouse. The experimental unit was the individual mouse. Ten mice were used in total and were allocated to the sh-NC and sh-CEACAM7 groups (*n* = 5 per group); no formal randomisation or blinding was applied. No anesthesia was required for the subcutaneous injection or for the external caliper measurement, and the animals were handled minimally and returned to their cage immediately afterward. Tumor volume was measured every 7 days with digital calipers (Mitutoyo, Kawasaki, Japan), and the general condition of the animals was monitored at the same time points. After four weeks, all mice were euthanized by carbon dioxide (CO_2_) inhalation, and the tumors were excised, weighed, and photographed. We snap-froze tumor tissues in liquid nitrogen for subsequent WB. No animals were excluded from the analysis.

### 2.13. Statistical Analysis

GraphPad Prism 8.0 and R (v4.2.1) were utilized for statistical analyses. All quantitative data are reported as mean ± standard deviation (SD) from at least three independent trials. Between-group variations were assessed via Student’s *t*-test. Specifically, a paired Student’s *t*-test was utilized to compare tumor tissues and matched neighboring healthy tissues, while an unpaired Student’s *t*-test was used for comparisons between two groups (e.g., cell line experiments or non-paired tissue samples). To compare several groups, we conducted a one-way analysis of variance (ANOVA). Specifically, Dunnett’s post hoc test was utilized for comparing several experimental groups against a single control group, while Tukey’s post hoc test was used for pairwise comparisons. *p* < 0.05 was deemed significant. The value of *n* reported in each figure legend refers to the number of independent biological experiments performed on separate occasions, rather than to technical replicates of the same sample. Both post hoc tests (Dunnett’s and Tukey’s) control the family-wise error rate for multiple comparisons.

## 3. Results

### 3.1. CEACAM7 Is Upregulated in LUAD and Triggers Tumor Cell Growth

**Figure 1:**
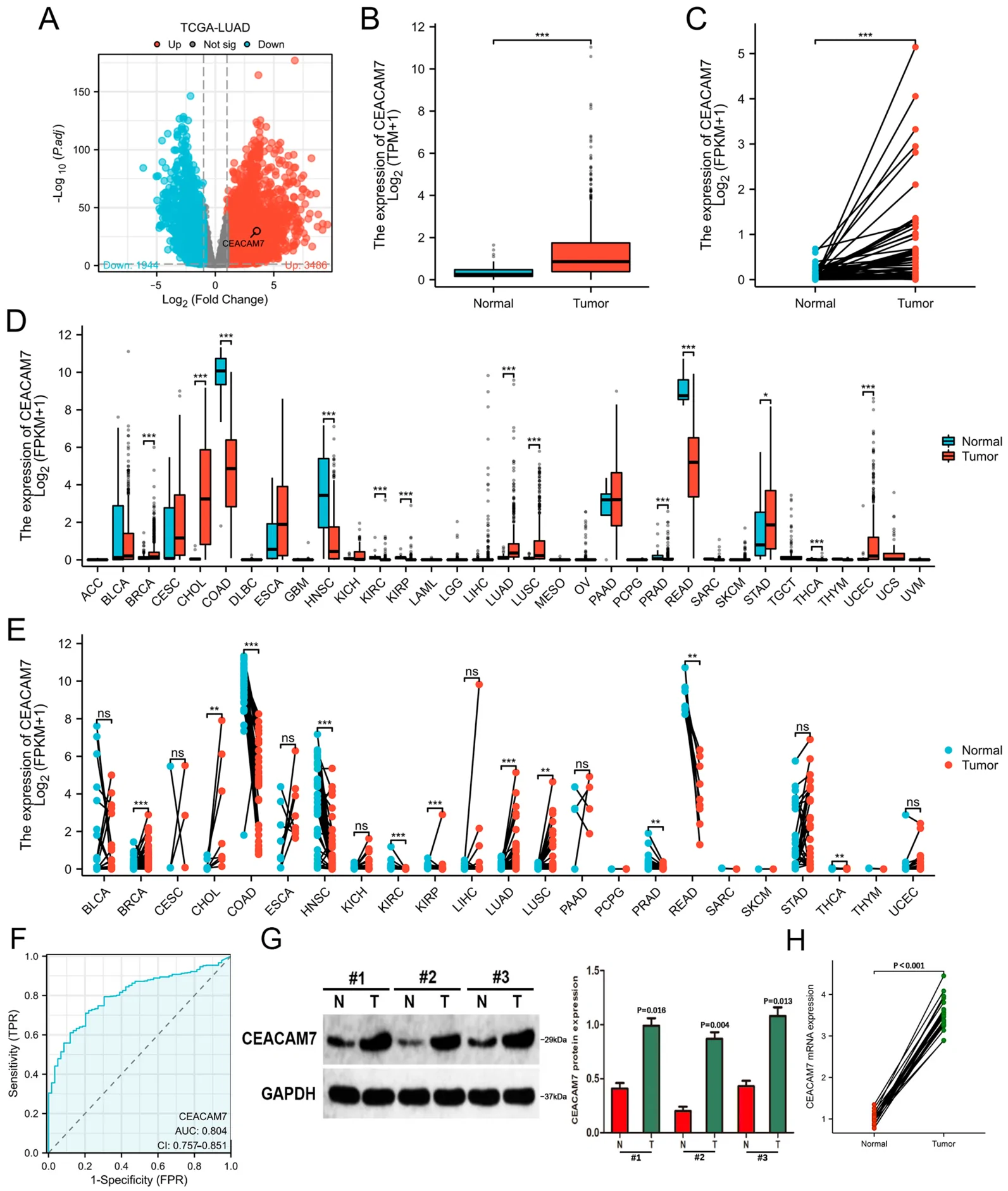
CEACAM7 is overexpressed in lung adenocarcinoma (LUAD). (**A**) Volcano plot of differentially expressed genes (DEGs), with overexpressed and downexpressed genes presented in red and blue, respectively. (**B**,**C**) CEACAM7 levels in LUAD versus normal tissues (**B**) and paired normal tissues (**C**). (**D**,**E**) Pan-cancer analysis of CEACAM7 expression in tumor vs. normal tissues (**D**) and paired non-tumor tissues (**E**). (**F**) ROC curve estimating the diagnostic capability of CEACAM7 in the TCGA-LUAD cohort (AUC = 0.804). (**G**,**H**) Validation of CEACAM7 overexpression in LUAD and neighboring non-malignant tissues by Western blot (WB) (**G**) and RT-qPCR (**H**). In (**H**), each symbol represents an individual patient; the samples are the same 27 paired LUAD and adjacent normal tissues collected at Punan Hospital, Pudong New Area, Shanghai, between March 2023 and March 2024, and paired samples are connected by a line. Numbers preceded by # denote individual patients; paired tumour and adjacent normal samples from the same patient carry the same number. Data are mean ± SD, three independent trials. * *p* < 0.05, ** *p* < 0.01, *** *p* < 0.001; ns, not significant.

**Figure 2:**
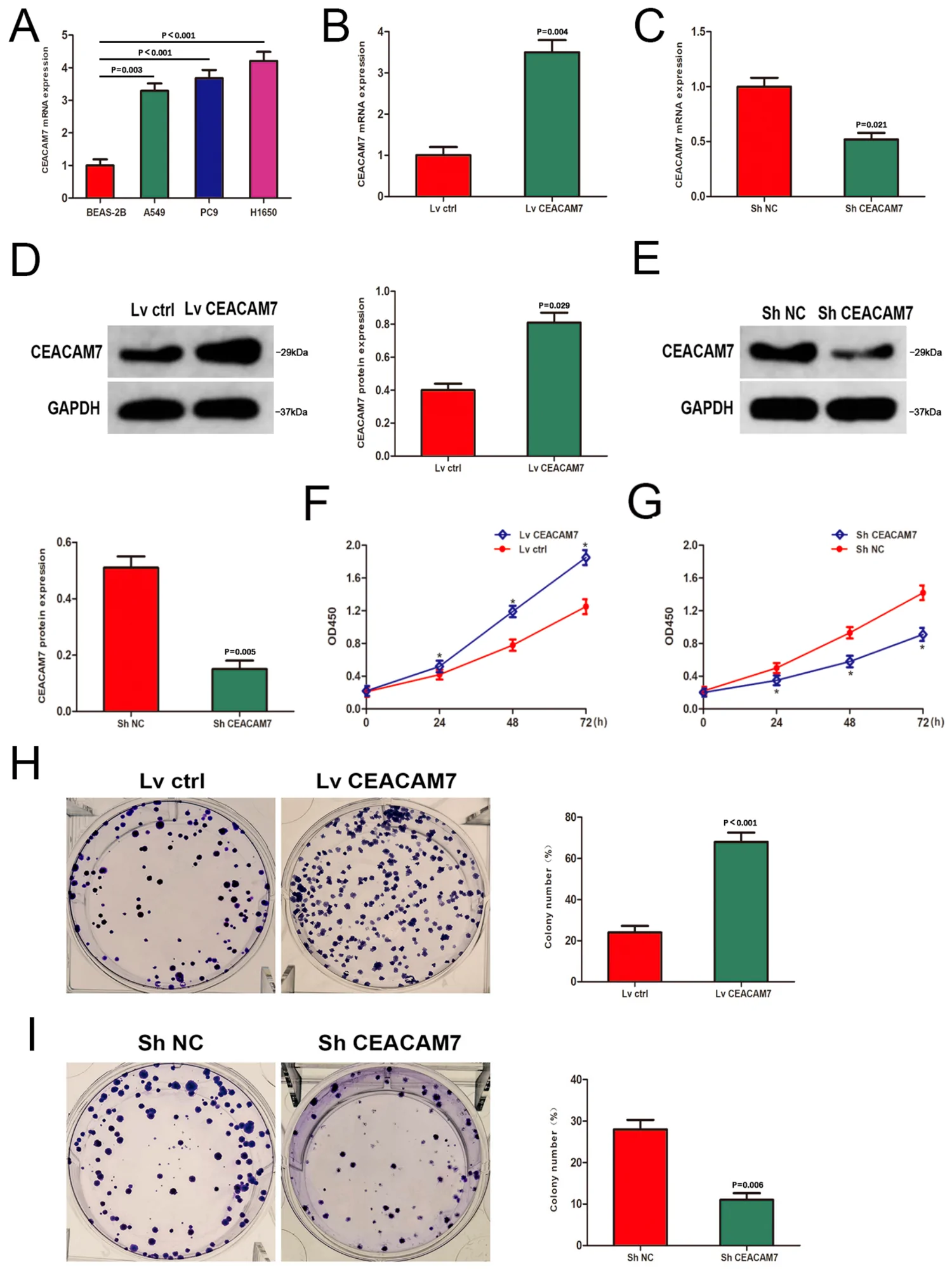
CEACAM7 is upregulated in LUAD cells and triggers cell growth. (**A**) RT-qPCR of CEACAM7 expression in BEAS-2B, A549, PC9, and H1650 cells. (**B**–**E**) Validation of CEACAM7 overexpression and knockdown by RT-qPCR and WB. (**F**,**G**) CCK-8 assays evaluating the effects of CEACAM7 knockdown or overexpression on H1650 cell proliferation over 72 h. (**H**,**I**) Colony formation assays estimating the influence of CEACAM7 downregulation or upregulation on the proliferative capability of H1650 cells. Data are mean ± SD from three independent trials. Statistical analysis was conducted via one-way ANOVA with Dunnett’s post hoc test (**A**) and Student’s *t*-test (**B**–**I**). * *p* <0.05.

First, we determined 5430 genes that showed differential expression in the context of the TCGA-LUAD project, meeting the criteria of |logFC| > 1 and adj *p*< 0.05. Among these differentially expressed genes, we observed that 1944 were characterized by low expression levels, whereas 3486 exhibited high expression levels (Figure 1A). To determine prospective therapeutic targets within the CEACAM family, we analyzed the expression profiles of all family members in the TCGA-LUAD dataset. Our analysis revealed distinct expression patterns: consistent with previous reports, CEACAM1, CEACAM5, and CEACAM6 were significantly upregulated in tumor tissues. Conversely, CEACAM3 and CEACAM4 showed low expression or downregulation (Supplementary Figure S1A–E). Notably, CEACAM7 exhibited significant upregulation comparable to the well-known oncogenic members. Therefore, CEACAM7 was selected for further investigation because, unlike the extensively characterized CEACAM1, 5, and 6, its specific function in LUAD progression remains largely unexamined. Further, it was observed that there was a notable elevation of CEACAM7 levels in LUAD tissues compared to healthy lung tissues (Figure 1B,C). To further validate these findings in an independent cohort, we analyzed the GSE118370 dataset from the GEO database. Consistent with the TCGA outcomes, compared to normal controls, CEACAM7 was significantly overexpressed in LUAD tissues (Supplementary Figure S1F). The findings illustrated that CEACAM7 was significantly overexpressed in various malignancies compared to corresponding control tissues (Figure 1D,E). The predictive efficiency of CEACAM7 for LUAD was estimated via ROC analysis, with an estimated area under the curve (AUC) of 0.804 (Figure 1F). Kaplan–Meier analysis of the TCGA-LUAD cohort further showed that patients with high CEACAM7 expression had shorter overall survival and disease-specific survival than those with low expression (Supplementary Figure S1G,H). WB illustrated a significant elevation in the protein level of CEACAM7 in LUAD samples compared to their corresponding control lung tissues (Figure 1G). The RT-qPCR findings revealed a marked elevation in CEACAM7 expression levels within malignant tissues as compared to their non-neoplastic counterparts (Figure 1H). Next, we examined CEACAM7 expression in LC cell lines (A549, PC9, H1650) and normal human lung epithelial cells (BEAS-2B) by RT-qPCR. The outcomes illustrated that CEACAM7 was significantly overexpressed in all LC cell lines compared to BEAS-2B cells, with the highest level observed in H1650 cells (Figure 2A). The same pattern was confirmed at the protein level by Western blotting (Supplementary Figure S2A). Based on these findings, H1650 cells were primarily chosen because they exhibited the highest endogenous CEACAM7 expression among the tested lines, making them the most appropriate model to estimate the effects of loss-of-function via shRNA knockdown. We also performed overexpression assays in these cells to investigate whether further elevating CEACAM7 levels would exacerbate malignant behaviors within the same genetic background. WB and RT-qPCR assays confirmed the effectiveness of CEACAM7 overexpression and knockdown, as shown in Figure 2B–E. To examine the influence of CEACAM7 on cell growth, CCK-8 and colony formation assays were conducted. The CCK-8 results illustrated that H1650 cells with CEACAM7 overexpression demonstrated significantly higher cell survival rates compared to the vector group. Conversely, compared with the sh-NC group, cell survival in the sh-CEACAM7 group was noticeably reduced (Figure 2F,G). Similarly, our colony formation assays yielded consistent results, demonstrating that CEACAM7 overexpression notably enhanced cell growth and proliferation, whereas CEACAM7 down-regulation hindered clone formation in vitro (Figure 2H,I). Collectively, these outcomes strongly illustrate that CEACAM7 has a pivotal function in triggering the proliferation of H1650 cells.

### 3.2. CEACAM7 Improves Cell Migration and Invasion of H1650 Cells

**Figure 3:**
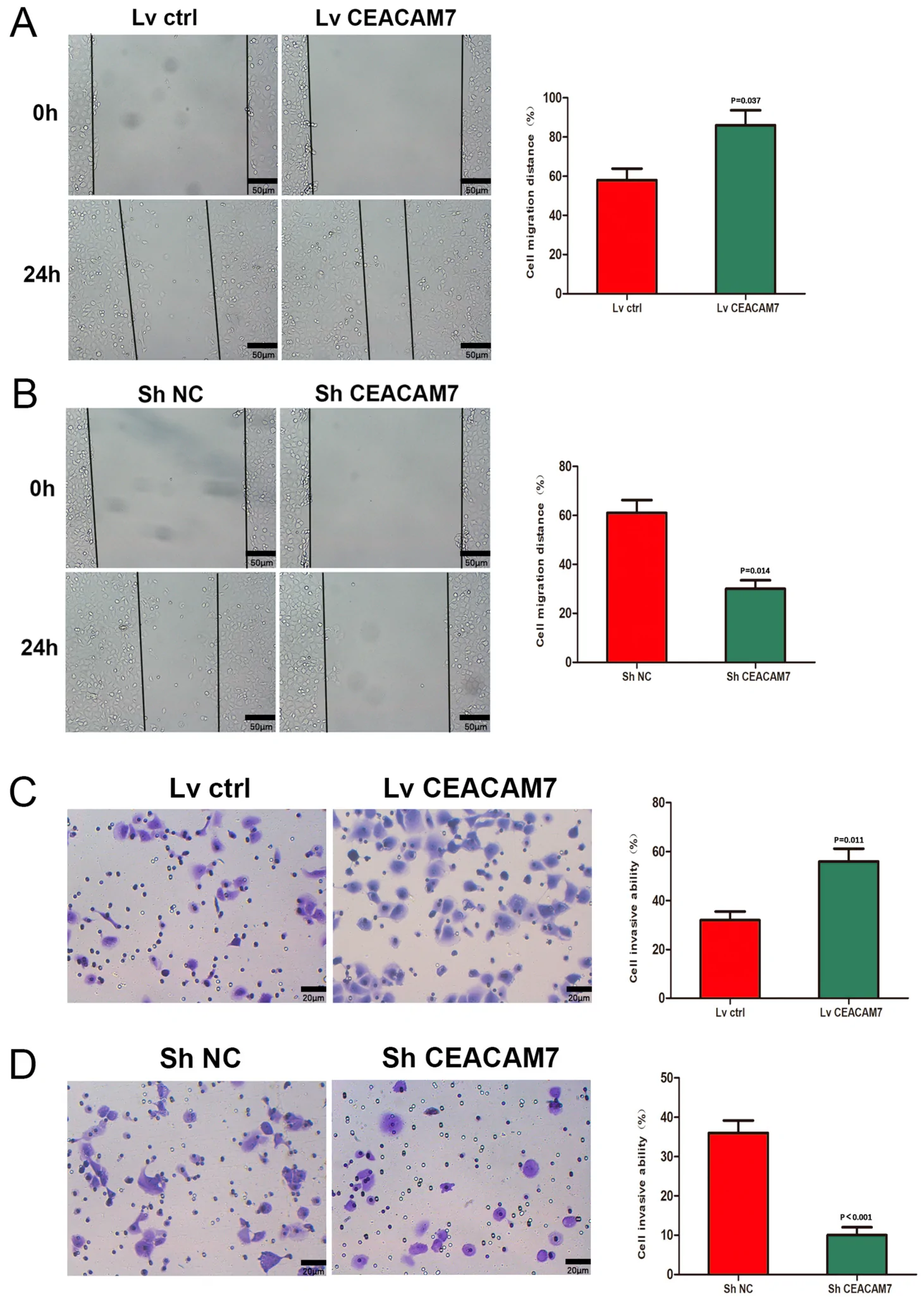
CEACAM7 promotes H1650 cell migration and invasion. (**A**,**B**) Wound-healing assays and quantification of cell migration at 0 and 24 h following CEACAM7 knockdown or overexpression. (**C**,**D**) Representative Transwell invasion assays and quantification of invasive capacity in H1650 cells; scale bars, 20 μm. Data are mean ± SD, three independent trials.

Subsequent experiments utilizing the H1650 cell line aimed to assess the influence of CEACAM7 on migratory invasion. The cell scratch assay results demonstrated that heightened expression of CEACAM7 considerably augmented the migratory capability of H1650 cells, while the down-regulation of CEACAM7 significantly hindered their migratory capacity (Figure 3A,B). Moreover, our Transwell invasion assay results were in line with the cell scratch assay outcomes. Overexpression of CEACAM7 markedly facilitated the invasion of H1650 cells, while knockdown of CEACAM7 hindered invasion (Figure 3C,D). These experimental outcomes underline the crucial function of CEACAM7 in LC invasion and migration. The migratory and invasive assays were repeated in A549 cells, in which endogenous CEACAM7 expression is lower than in H1650, and the results were consistent (Supplementary Figure S2B–G).

### 3.3. CEACAM7 Regulates GCNT3 Expression in LUAD

**Figure 4:**
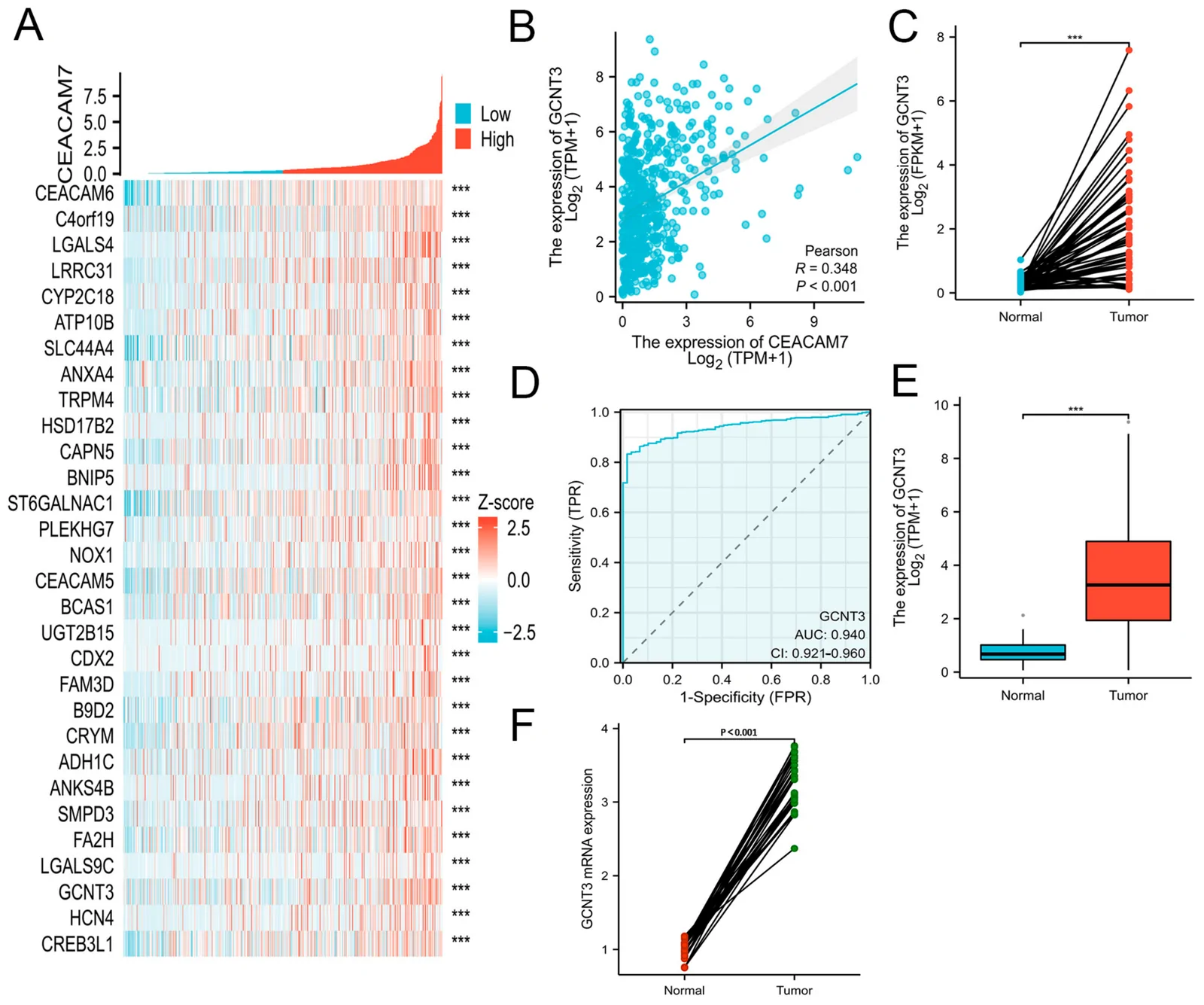
CEACAM7 is positively associated with GCNT3 expression in LUAD. (**A**) Heatmap of the top 30 genes positively related to CEACAM7 in the TCGA-LUAD dataset. (**B**) Correlation analysis showing a strong positive association between CEACAM7 and GCNT3 levels in LUAD. (**C**,**E**) GCNT3 expression in LUAD versus paired normal tissues (**C**) and unpaired normal tissues (**E**). (**D**) ROC curve estimating the diagnostic capability of GCNT3 in the TCGA-LUAD cohort (AUC = 0.940). (**F**) RT-qPCR of GCNT3 expression in LUAD and neighboring non-malignant tissues. In (**F**), each symbol represents an individual patient; the samples are the same 27 paired LUAD and adjacent normal tissues collected at Punan Hospital, Pudong New Area, Shanghai, between March 2023 and March 2024, and paired samples are connected by a line. Comparisons were made using a paired Student’s *t*-test. Data are mean ± SD, three independent trials. *** *p* <0.001.

**Figure 5:**
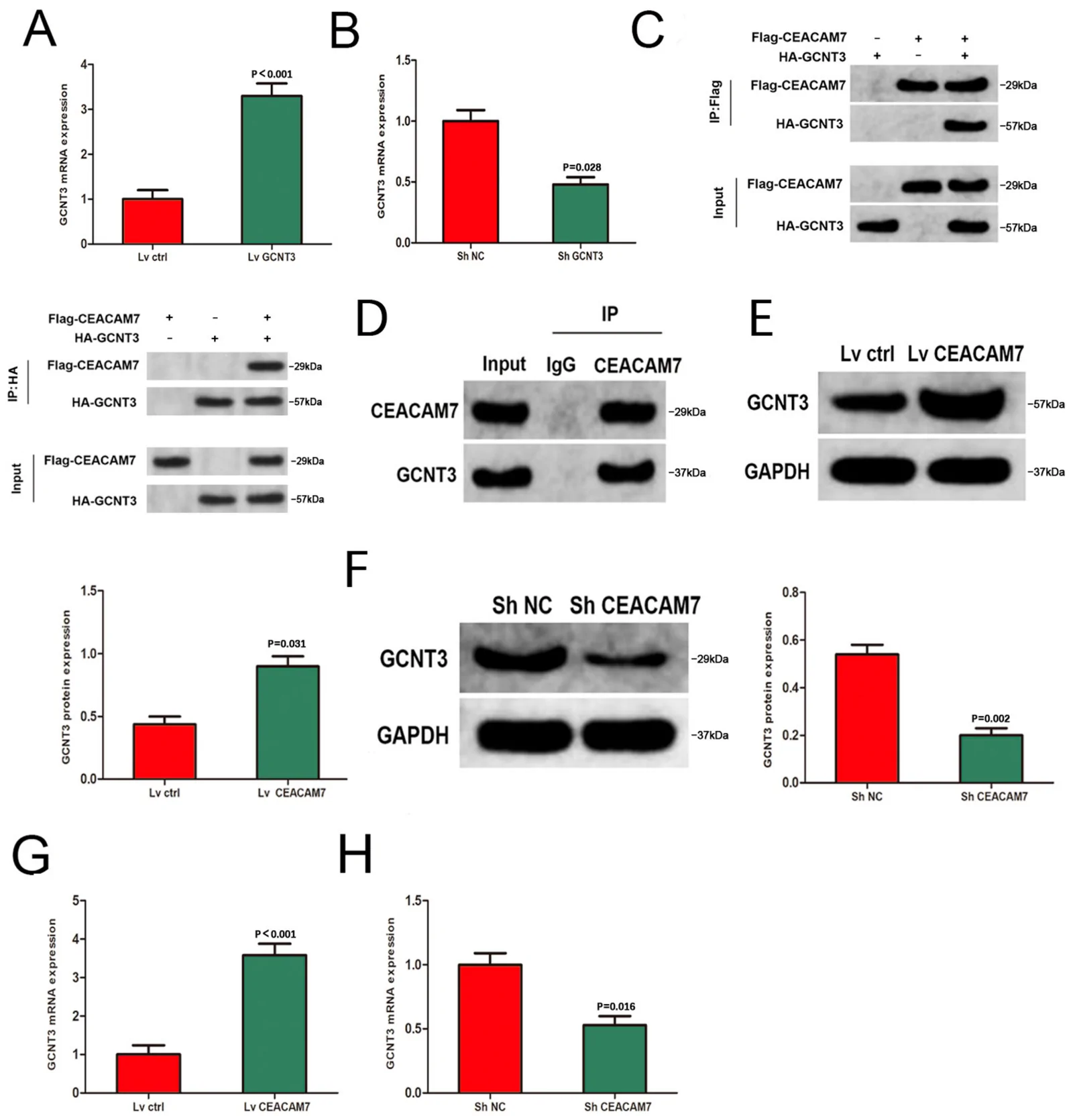
CEACAM7 controls GCNT3 expression in H1650 cells. (**A**,**B**) Validation of GCNT3 overexpression and knockdown by RT-qPCR. (**C**) Co-immunoprecipitation assays showing the interaction between exogenous CEACAM7 and GCNT3 in HEK293T cells in both the forward (anti-Flag) and reverse (anti-HA) directions, with an IgG control. (**D**) Co-immunoprecipitation of endogenous CEACAM7 and GCNT3 in H1650 cells. (**E**–**H**) RT-qPCR and WB analyses of GCNT3 expression subsequent to CEACAM7 overexpression or suppression in H1650 cells. Data are mean ± SD, three independent trials.

To improve our comprehension of the possible functions and molecular mechanisms of CEACAM7 in LUAD, we employed data from the TCGA database to create heatmaps showcasing the top 30 genes that showed a strong positive relationship with CEACAM7 in LUAD (Figure 4A). We conducted an analysis to examine the association between CEACAM7 and GCNT3 levels in TCGA-LUAD patient data, and our outcomes illustrated a significant positive relationship between these two genes in LUAD (Figure 4B). According to the TCGA data, a significant elevation in GCNT3 levels was observed in LUAD tissues (Figure 4C,E). The predictive effectiveness of GCNT3 for LUAD was estimated via ROC analysis, with an estimated AUC of 0.940 (Figure 4D). The RT-qPCR findings revealed a marked elevation in GCNT3 levels within malignant tissues as compared to their non-neoplastic counterparts (Figure 4F). In order to delve deeper into the association between GCNT3 and CEACAM7, we conducted overexpression and knockdown experiments targeting the GCNT3 gene in the H1650 cell line. The efficacy of the upregulation and downregulation procedures was estimated through qRT-PCR analysis (Figure 5A,B). To validate whether CEACAM7 directly interacts with GCNT3, we performed Co-IP assays. First, we utilized HEK293T cells for the exogenous Co-IP assay, in which the interaction was examined in both the forward (anti-Flag) and reverse (anti-HA) directions (Figure 5C). HEK293T cells were chosen for this trial because of their exceptionally high transfection efficacy and capability to express exogenous proteins at robust levels, which ensures sufficient bait and prey protein abundance for the clear detection of physical interactions. Subsequently, to validate this interaction in a physiological context, we confirmed the CEACAM7-GCNT3 interaction by endogenous Co-IP assay detection in H1650 cells (Figure 5D). Furthermore, RT-qPCR and WB outcomes illustrated that CEACAM7 upregulation significantly increased GCNT3, whereas suppression of CEACAM7 downregulated the expression level of GCNT3 (Figure 5E–H).

### 3.4. CEACAM7 Promotes Malignant Biological Behavior of LUAD Cells Through GCNT3

**Figure 6:**
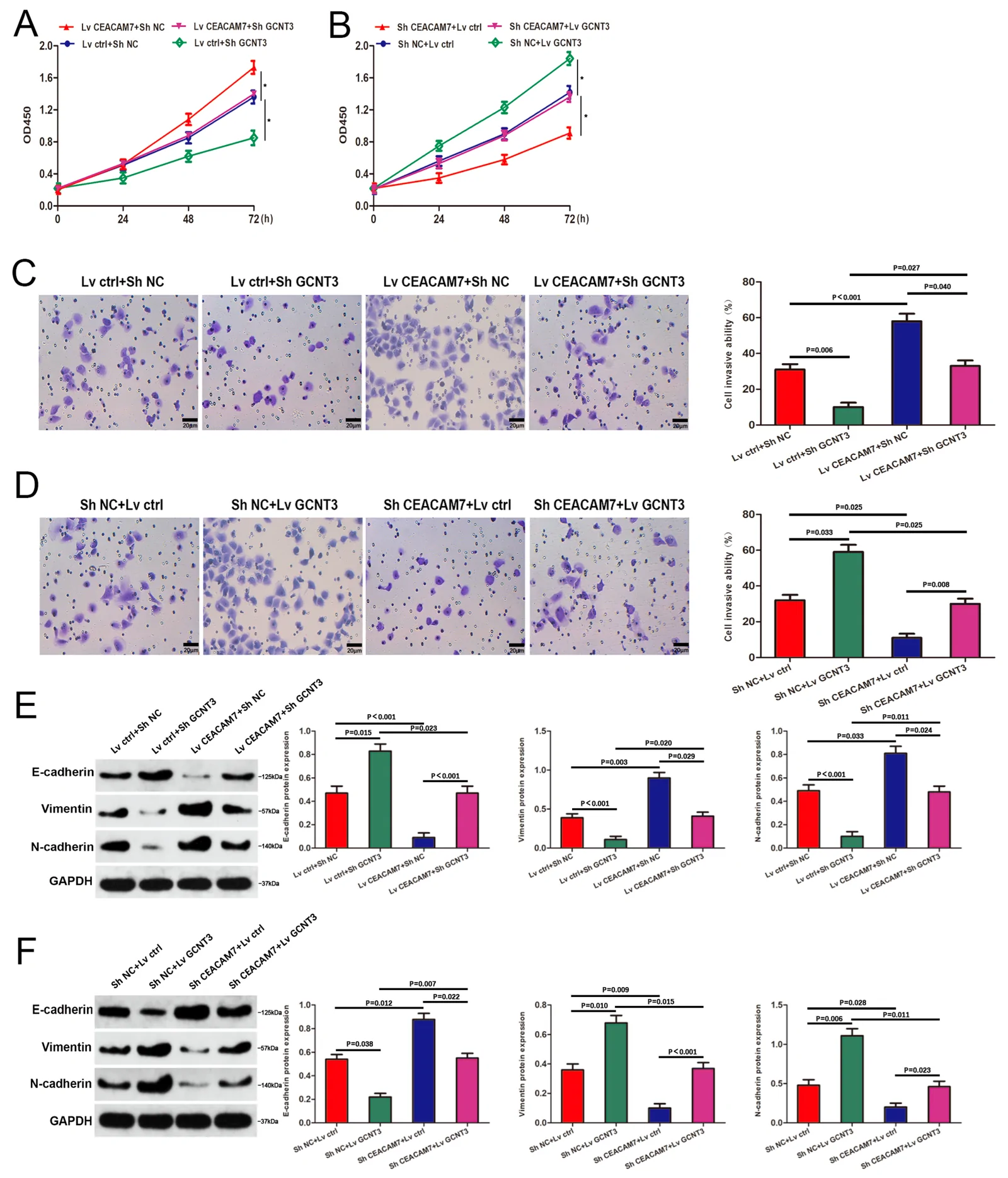
CEACAM7 promotes malignant phenotypes of LUAD cells through GCNT3. (**A**,**B**) CCK-8 assays assessing proliferation of H1650 cells across experimental groups. (**C**,**D**) Matrigel-coated Transwell assays evaluating invasive capacity; scale bars, 20 μm. (**E**,**F**) WB of EMT-related protein levels. Data are mean ± SD, three independent trials. Statistical significance among multiple groups was estimated via one-way ANOVA and then Tukey’s post hoc test. * *p* <0.05.

After identifying the regulatory relationship between CEACAM7 and GCNT3, we further examined the function of the CEACAM7-GCNT3 axis in the biological function of LUAD cells. The proliferative viability of each group of cells was observed by CCK-8, and GCNT3 silencing reversed the elevation in cell proliferation triggered by CEACAM7 upregulation, whereas GCNT3 overexpression reversed the decrease in cell proliferation triggered by CEACAM7 silencing (Figure 6A,B). Transwell analysis also showed the same results, with silencing of GCNT3 reversing the increase in cell invasion resulting from CEACAM7 overexpression, whereas overexpression of GCNT3 reversed the decrease in cell invasion resulting from silencing of CEACAM7 (Figure 6C,D). WB showed that silencing of GCNT3 reversed the decrease in cellular E-cadherin protein expression and the elevation of Vimentin and N-cadherin expression caused by overexpression of CEACAM7, whereas GCNT3 overexpression reversed the E-cadherin overexpression and the Vimentin and N-cadherin downregulation caused by silencing of CEACAM7 (Figure 6E,F). Furthermore, we observed that the levels of EMT-inducing transcription factors, Slug and ZEB1, were positively regulated by CEACAM7 (Supplementary Figure S3A,B). Taken together, these outcomes illustrate that CEACAM7 triggers the growth, invasion and EMT capacity of H1650 cells by upregulating GCNT3 expression.

### 3.5. CEACAM7 Promotes LUAD Tumor Growth and EMT In Vivo

**Figure 7:**
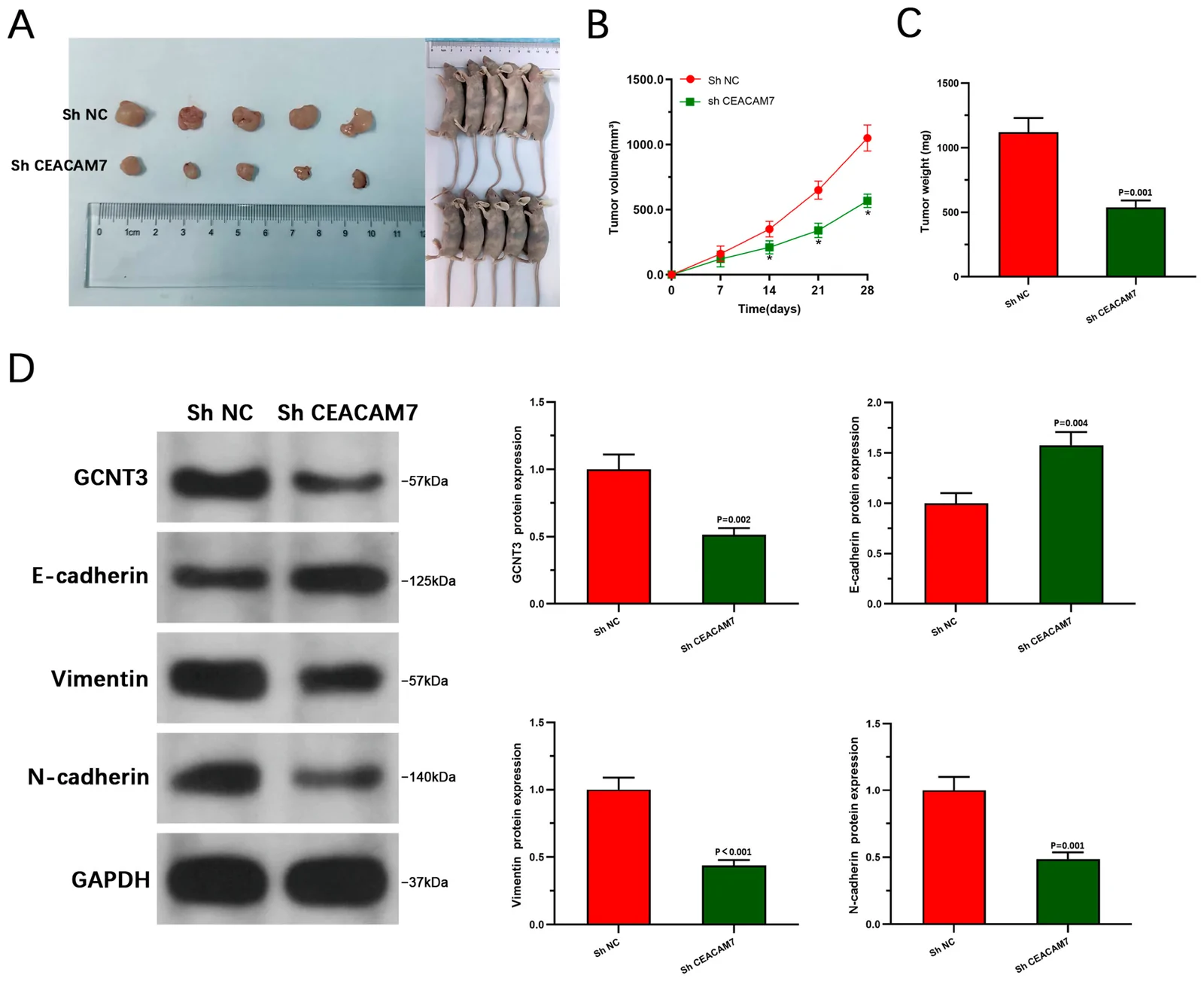
CEACAM7 knockdown suppresses LUAD tumor growth and reverses EMT in vivo, with reduced GCNT3 expression. (**A**) Images of subcutaneous xenograft tumors from nude mice injected with H1650 cells expressing sh-NC or sh-CEACAM7. (**B**) Tumor growth curves displaying alterations in tumor volume over time. (**C**) Tumor weights at endpoint. (**D**) WB analysis of GCNT3 and EMT-related markers in xenograft tissues. Data are mean ± SD (*n* = 5/group). * *p* < 0.05.

To verify the oncogenic function of CEACAM7 in LUAD in vivo, we conducted a xenograft tumor model in nude mice. H1650 cells stably transfected with sh-NC or sh-CEACAM7 were introduced via subcutaneous injection into the right flank of nude mice. Figure 7A illustrates that the tumor size in the sh-CEACAM7 group was visibly decreased compared to the sh-NC group at the end of the trial. We regularly measured the tumor dimensions to calculate the tumor volume, and the growth curve demonstrated that, compared to the control, CEACAM7 downregulation significantly suppressed tumor growth (Figure 7B). Consistently, compared with the sh-NC group, the final tumor weight in the sh-CEACAM7 group was markedly decreased (Figure 7C). Furthermore, we isolated proteins from the excised tumor tissues and conducted WB to evaluate the levels of GCNT3 and EMT-related markers. The outcomes revealed that the protein GCNT3, N-cadherin, and Vimentin levels were significantly downregulated in the sh-CEACAM7 group, whereas the levels of E-cadherin were notably upregulated (Figure 7D). Collectively, these in vivo findings show that CEACAM7 knockdown suppresses tumor growth and reverses EMT in xenografts, and that this is accompanied by reduced GCNT3 expression. Because this experiment compared only the sh-NC and sh-CEACAM7 groups, the causal role of GCNT3 in vivo is not established here.

## 4. Discussion

As per the latest global cancer statistics, LC is still the main cause of cancer-related mortalities, representing 18% of all cancer mortalities globally [1]. In China, LC is considered one of the factors yielding high cancer-related mortality, accounting for 19% [19]. This phenomenon may be related to the increase in the number of LC patients among young smokers. Recently, with the development of medical technology, treatment strategies (e.g., surgery, radiotherapy, chemotherapy) and targeted therapy have made great progress, and the prognosis of LC patients has also improved. Nevertheless, the prognosis of LC patients is still unsatisfactory, particularly those with metastasis, recurrence and drug resistance. Thus, it is very important to examine the new mechanisms and targets of radiotherapy resistance in LUAD [20,21,22]. CEACAM7 is a part of the CEA family. CEACAMs, parts of the Ig family, are differentially expressed on the cell surface. Functional cellular processes of CEACAMs are very diverse, such as cell adhesion, tumor growth, progression, signaling and phagocytosis [13]. CEACAM7 is a kind of protein which is rarely studied. It has been studied only in colorectal cancer and pancreatic cancer, but not in other tumors [23].

Nevertheless, the biological function of CEACAM7 in LC and its mechanisms are still unknown. Herein, using data from the TCGA database, we discovered that the CEACAM7 level in LC tissues was significantly higher than that in neighboring non-cancerous tissues, which was further validated by clinical samples. In addition, the bioinformatics analysis data illustrated that CEACAM7 may have a function in the progression of LUAD, which prompted further studies using H1650 cells as an in vitro experimental model. The outcomes illustrated that up-regulation of CEACAM7 triggered aggressiveness and EMT of H1650 cells, while the downregulation of CEACAM7 played the opposite role.

GCNT3 (Glucosaminyl (N-acetyl) transferase3) is a mucin-type synthetase that has a vital function in glycan synthesis [24]. Earlier investigations have illustrated that GCNT3 has a vital function in various malignancies (e.g., breast, prostate, colon, gastric, and liver cancers). Several investigations have illustrated that high GCNT3 expression enhances cell aggressiveness [25,26,27,28]. To elucidate the molecular mechanisms driving CEACAM7-mediated progression, we investigated its relationship with GCNT3. We observed a direct interaction between CEACAM7 and GCNT3, alongside a positive correlation in clinical samples. Notably, our data revealed that CEACAM7 overexpression significantly upregulates GCNT3 at the mRNA and protein levels, although the level at which this regulation occurs has not been defined directly. Given that CEACAM7 is a GPI-anchored protein lacking an intracellular domain, we hypothesize that it functions by recruiting co-receptors to trigger downstream signaling cascades, which in turn modulate the transcription factors governing GCNT3 expression. A follow-up study utilizing promoter activity assays and ChIP-qPCR to map these specific signaling axes is being established. Functionally, we verified that GCNT3 is a critical downstream effector: suppression of GCNT3 reversed the improved aggressiveness triggered by CEACAM7 upregulation, whereas GCNT3 restoration rescued the suppressive effects of CEACAM7 knockdown. Collectively, these findings indicate that CEACAM7 promotes LUAD development through the GCNT3 axis and represents a potential therapeutic target.

Mechanistically, the interaction between CEACAM7 and GCNT3 provides a novel perspective on how surface adhesion molecules regulate intracellular glycosylation machinery. GCNT3 is a key glycosyltransferase responsible for core 2 O-glycan synthesis, which is critical for the stability and function of numerous glycoproteins (e.g., mucins and integrins) [29]. Aberrant O-glycosylation is known to disrupt cell–cell adhesion and promote metastasis by modulating integrin signaling pathways [30]. In our study, the upregulation of GCNT3 by CEACAM7 likely leads to altered glycosylation of cell surface receptors, thereby activating downstream signaling cascades that drive EMT. This is consistent with our observation of altered E-cadherin and Vimentin levels. We hypothesize that CEACAM7 may act as a scaffold protein, recruiting GCNT3 to specific membrane domains to facilitate the glycosylation of integrins or other adhesion molecules, ultimately enhancing the invasive potential of LUAD cells.

Recent investigations have presented insights into the signaling mechanisms by which CEACAM7 affects gene expression. For instance, in nasopharyngeal carcinoma, CEACAM7 was reported to trigger cell migration and upregulate BST2 via the JAK2/STAT3 signaling axis [31]. Likewise, in oral malignancy, CEACAM7 has been shown to enhance metastasis through the stimulation of p-JNK and p-Src pathways, which subsequently regulate CD317 expression [32]. These findings indicate that CEACAM7, despite being a GPI-anchored protein, is able to recruit and activate intracellular kinases that modulate downstream gene transcription, which is consistent with the positive association between CEACAM7 and GCNT3 observed in the present study.

Current therapeutic strategies for LUAD have evolved significantly, with molecular targeted therapies (e.g., EGFR-TKIs like Gefitinib and Osimertinib) and immunotherapies (e.g., PD-1/PD-L1 inhibitors) becoming standard treatments for advanced cases [33]. Nevertheless, the efficiency of these therapies is often compromised by developing drug resistance. Recent investigations have underlined the contribution of CEACAM family members in these resistance mechanisms. For instance, CEACAM6 overexpression has been reported to confer resistance to EGFR-TKIs in LUAD cells by sustaining p-ERK1/2 signaling and promoting EMT [12]. Additionally, CEACAM5 is currently being examined as a promising target for antibody-drug conjugates (ADCs) clinically for non-squamous NSCLC [34]. In this context, our finding that CEACAM7 is upregulated in LUAD and promotes tumorigenesis via the GCNT3 axis suggests that it could act as a novel therapeutic target, potentially presenting a novel avenue for patients who are unresponsive or resistant to existing CEACAM-targeted or EGFR-targeted therapies.

From a clinical perspective, the cell-surface localization and restricted expression pattern of CEACAM7 make it an attractive target for precision therapy. Unlike intracellular proteins that are difficult to target, CEACAM7 is accessible to antibody-based therapeutics. Potential strategies could include the development of monoclonal antibodies or ADCs that specifically bind to CEACAM7 to deliver cytotoxic agents directly to tumor cells [35]. Furthermore, recent studies have demonstrated the effectiveness of CEACAM7-targeted CAR-T cell therapy in pancreatic cancer, suggesting a similar approach could be explored for LUAD patients with high CEACAM7 expression [23]. Small molecule inhibitors targeting the interaction interface between CEACAM7 and GCNT3 could also represent a novel therapeutic avenue to block the downstream oncogenic signaling without affecting normal tissues.

Although there are promising outcomes, there are various limitations to this investigation. First, although we utilized the large-scale TCGA dataset for bioinformatic analysis, our experimental validation was conducted on a relatively small clinical cohort (*n* = 27). While the clinical data were consistent with the bioinformatics results, the small sample size may present selection bias. Upcoming investigations utilizing larger, multi-center independent cohorts are required to verify the diagnostic and predictive value of CEACAM7 in LUAD. Second, the in vivo experiments were conducted in a single immunodeficient mouse strain using subcutaneous, rather than orthotopic, implantation; this model does not reproduce the native pulmonary microenvironment or the anti-tumor immune response, and the absence of formal randomisation and blinding may introduce bias. Therefore, the in vivo findings should be extrapolated to human LUAD with caution, and validation in immunocompetent or patient-derived xenograft models will be required. Third, the in vivo experiment compared only the sh-NC and sh-CEACAM7 groups, and therefore establishes the effect of CEACAM7 knockdown on tumor growth without identifying GCNT3 as the mediator. An in vivo rescue experiment, in which GCNT3 is re-expressed in CEACAM7-silenced cells, will be required to establish causality, and is planned as the immediate next step. Fourth, the molecular mechanism by which CEACAM7 influences GCNT3 expression remains undefined. Because CEACAM7 is GPI-anchored and does not bind DNA, resolving this will require identifying the intermediate transcription factor(s) and testing their recruitment to the GCNT3 promoter by reporter and ChIP-qPCR assays, and this forms the basis of a follow-up study. Fifth, sex was not analysed as a biological variable: the xenograft experiment used female mice only, and although the clinical cohort was drawn from consecutive patients of both sexes, it was not powered to detect sex-specific differences.

## 5. Conclusions

Collectively, this study identifies CEACAM7 as a tumor-promoting cell-surface protein in LUAD. CEACAM7 was significantly overexpressed in LUAD tissues and correlated with tumor aggressiveness and poor prognosis, whereas its knockdown suppressed the malignant phenotype of H1650 cells in vitro and inhibited xenograft tumor growth in nude mice. CEACAM7 expression was positively associated with GCNT3, and the in vitro rescue experiments indicate that GCNT3 is required for the pro-tumorigenic effects of CEACAM7, although the level at which this regulation occurs remains to be defined. Given its cell-surface localisation and restricted expression in normal tissues, CEACAM7 warrants further evaluation as a diagnostic biomarker and as a candidate for antibody-based therapy in LUAD.

## Figures and Tables

**Table 1:** Primer sequences used for RT-qPCR.

Gene	Forward (5′-3′)	Reverse (5′-3′)
CEACAM7	GTTACCCACAATGACGCAGGA	TCCACCGGATTGAAGTTGTTG
GCNT3	TCAAAGAGGCGGTCAAAGCAA	GCATAAACCACCCGAACCAG
GAPDH	GTGGACCTGACCTGCCGTCTAG	GAGTGGGTGTCGCTGTTGAAGTC

## Data Availability

The original data presented in this study are available on request from the corresponding author due to patient privacy and institutional data-sharing restrictions. Publicly available datasets were analysed in this study; the TCGA-LUAD data can be found at https://portal.gdc.cancer.gov/, accessed on 10 March 2024 and the GSE118370 dataset at https://www.ncbi.nlm.nih.gov/geo/, accessed on 10 March 2024. The R code used for the bioinformatic analyses is available from the corresponding author on reasonable request. The Analysis Code presented in this study are included in the Supplementary Materials (Code S1).

## Supplementary Materials

The following supporting information can be downloaded at https://www.mdpi.com/article/10.3390/cancers18213486/s1, Figure S1: Expression profiles of CEACAM family members in LUAD; Figure S2: CEACAM7 expression in lung cancer cell lines and validation of CEACAM7 gain- and loss-of-function in A549 cells; Figure S3: CEACAM7 promotes malignant biological behavior of LUAD cells through GCNT3; File S1: Original Western blot images, together with densitometric quantification of the blots; Code S1: Analysis_Code_CEACAM7_GCNT3.
