# Supplementary material for: CEACAM7 Promotes Tumor Progression by Regulating GCNT3 Expression in Lung Adenocarcinoma
Source: Cancers (Basel). 2026 Oct 29;18(21):3486. doi: 10.3390/cancers18213486 (PMC13761727; doi:10.3390/cancers18213486)
Supplement: Supplementary file 1 [file cancers-18-03486-s001.zip › Figures.pdf]

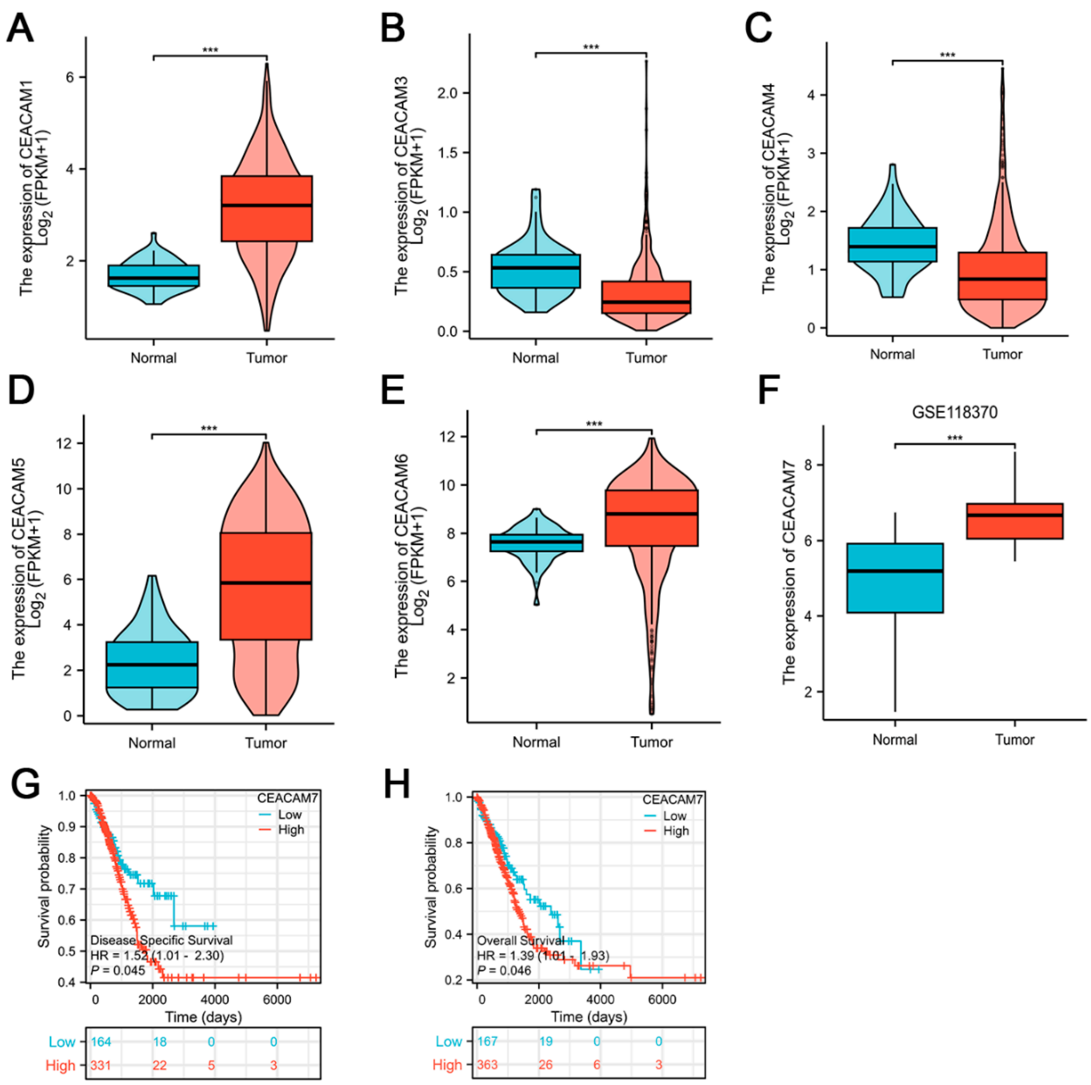

Supplementary Figure S1. Expression profiles of CEACAM family members in LUAD. (A-E) Analysis of the TCGA-LUAD dataset showing the differential expression levels of CEACAM1, CEACAM3, CEACAM4, CEACAM5, and CEACAM6 between LUAD tumor tissues and normal tissues. (F) Validation of CEACAM7 expression in an independent cohort using the GSE118370 dataset from the GEO database. (G, H) Kaplan–Meier analysis of overall survival and disease-specific survival by CEACAM7 expression. ***P < 0.001.

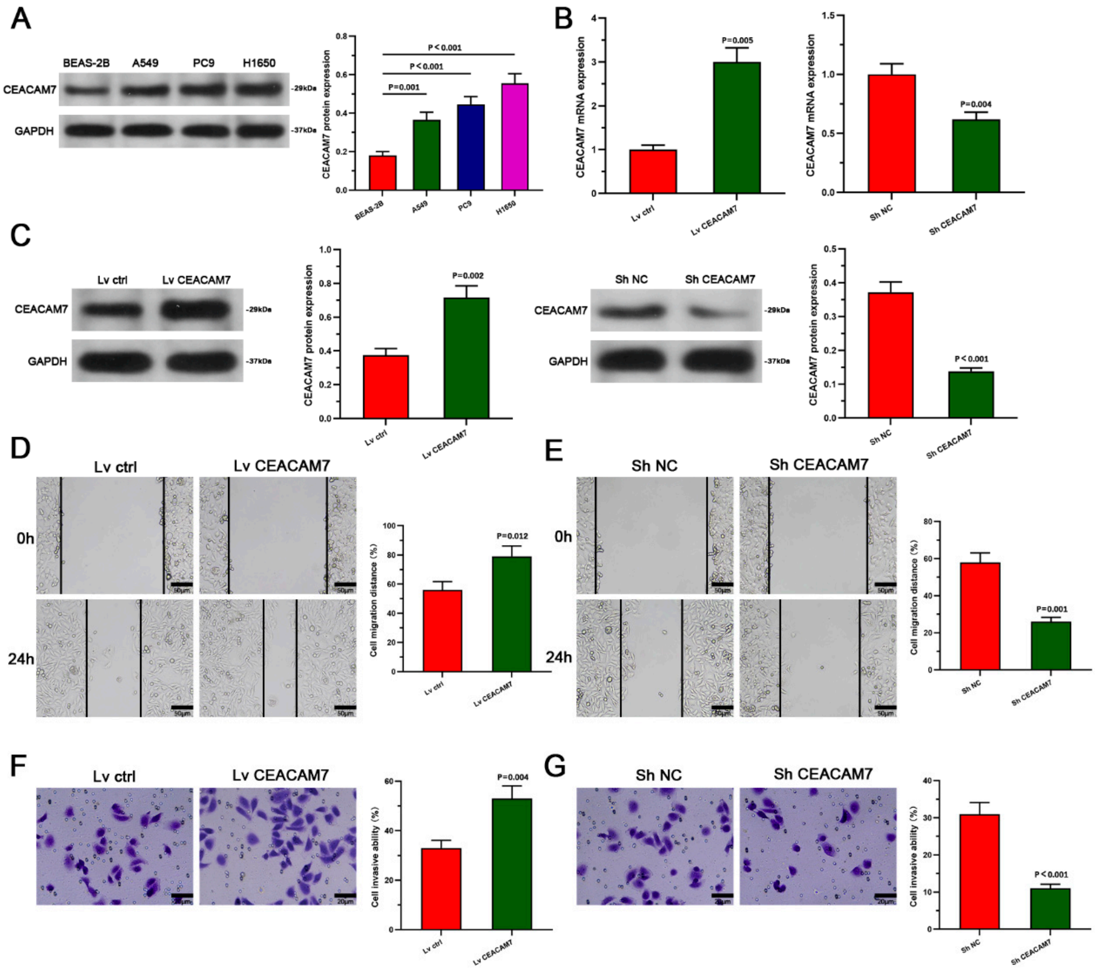
A
BEAS-2B
A549
PC9
H1650
CEACAM7
GAPDH
-29kDa
-37kDa
CEACAM7 protein expression
P＜0.001
P＜0.001
P=0.001
B
CEACAM7 mRNA expression
P=0.005
Lv ctrl
Lv CEACAM7
P=0.004
Sh NC
Sh CEACAM7
C
P=0.002
P＜0.001
D
E
0h
24h
Cell migration distance (%)
P=0.012
P=0.001
F
G
Cell invasive ability (%)
P=0.004
P＜0.001

Supplementary Figure S2. CEACAM7 expression in lung cancer cell lines and validation of CEACAM7 gain- and loss-of-function in A549 cells. (A) Western blot and quantification of CEACAM7 in BEAS-2B, A549, PC-9 and H1650 cells. (B, C) RT-qPCR (B) and western blot (C) validation of CEACAM7 overexpression and knockdown in A549 cells. (D, E) Wound-healing assay in A549 cells after CEACAM7 overexpression and knockdown. (F, G) Transwell invasion assay after overexpression and knockdown. Data are mean ± SD of three independent experiments; unpaired two-tailed t-test.

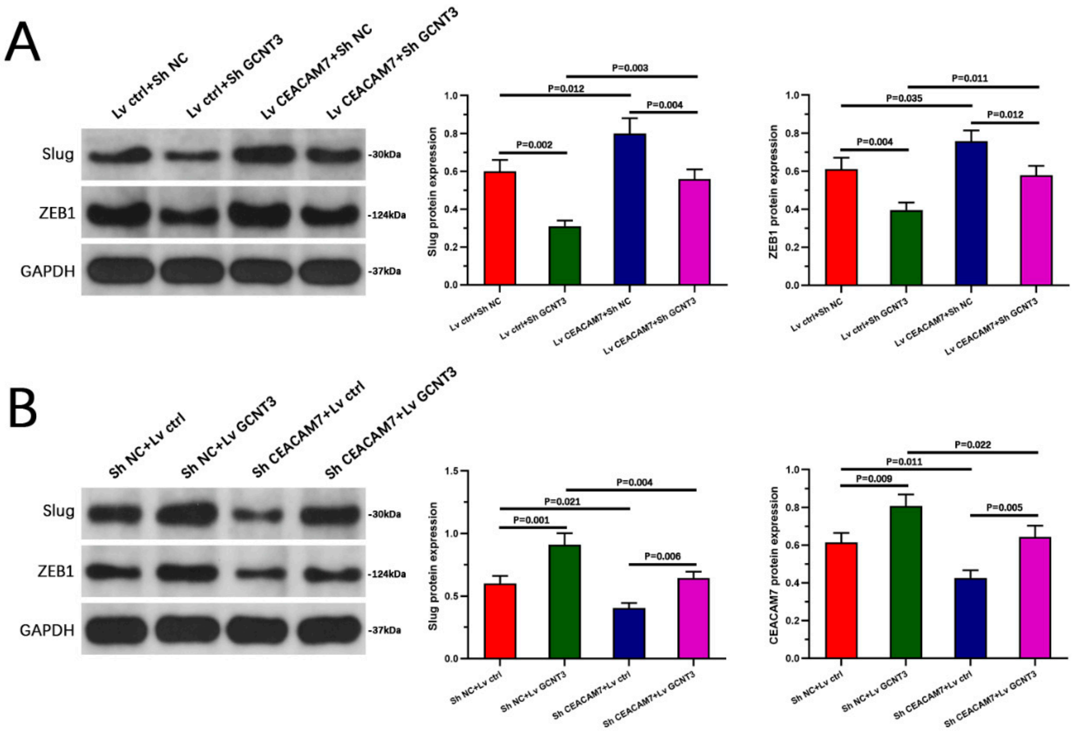

Supplementary Figure S3. CEACAM7 promotes malignant biological behavior of LUAD cells through GCNT3. (A, B) Western blotting assay was used to detect the protein expression levels of the EMT-inducing transcription factors Slug and ZEB1 in each group of cells. Data are presented as mean ± SD from three independent experiments. Statistical significance among multiple groups was determined using one-way ANOVA followed by Tukey's post hoc test.
