# Supplementary material for: CEACAM7 Promotes Tumor Progression by Regulating GCNT3 Expression in Lung Adenocarcinoma
Source: Cancers (Basel). 2026 Oct 29;18(21):3486. doi: 10.3390/cancers18213486 (PMC13761727; doi:10.3390/cancers18213486)
Supplement: Supplementary file 1 [file cancers-18-03486-s001.zip › File S1.pdf]

FIG.1 G CEACAM7

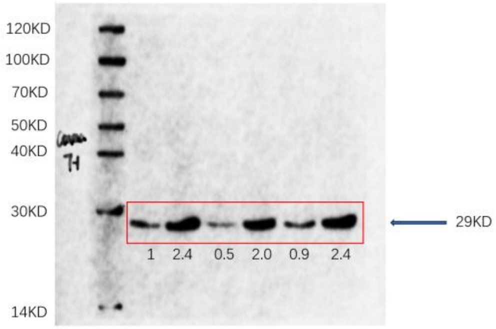

FIG.1 G GAPDH

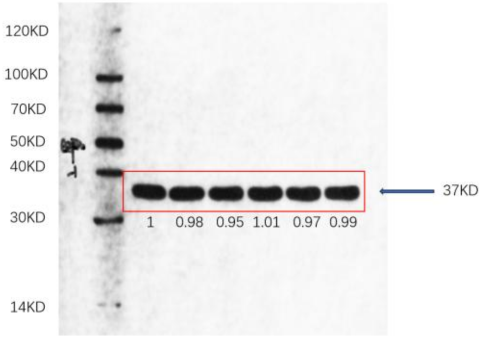

ceacam7	3304	7831	1486	6645	3140	7882
GAPDH	7976	7888	7586	7675	7363	7307
ceacam7/GAPDH	0.414	0.993	0.196	0.866	0.426	1.079

FIG.2 D CEACAM7

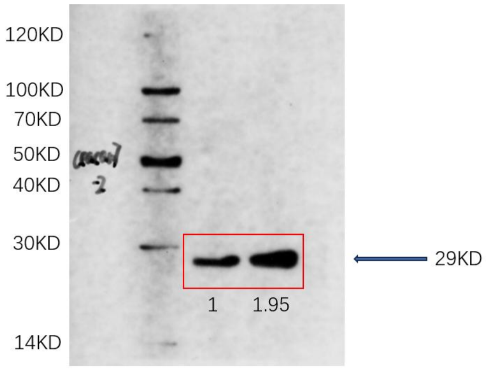

FIG.2 D GAPDH

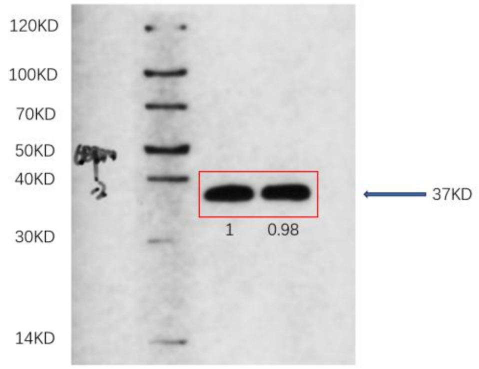

ceacam7	3970	7760
GAPDH	9828	9592
ceacam7/GAPDH	0. 404	0. 809

FIG.2 E CEACAM7

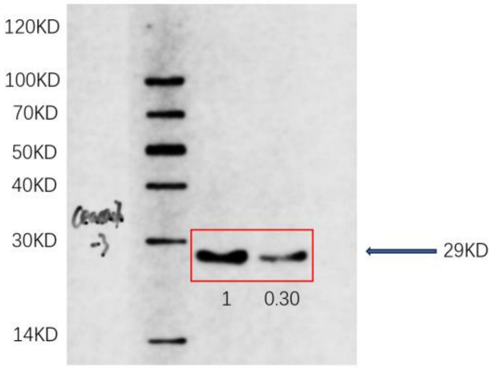

FIG.2 E GAPDH

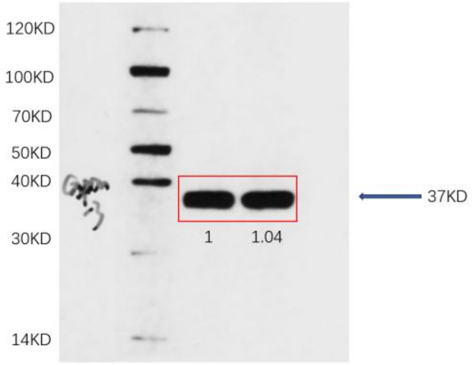

ceacam7	5914	1763
GAPDH	11569	12026
ceacam7/GAPDH	0. 511	0. 147

FIG.5 C FLAG-CEACAM7

The western blots from the Co-IP and pull-down assays only show that the two proteins bind to each other and do not require densitometric quantification.

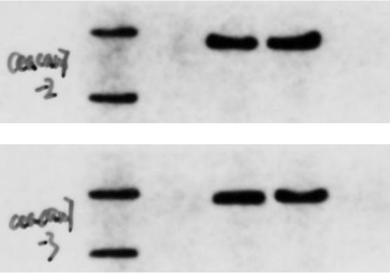

FIG.5 C HA-GCNT3

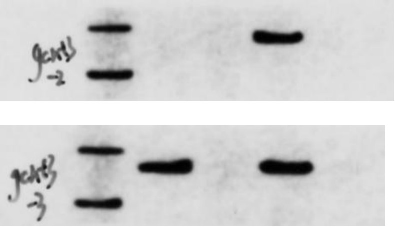

FIG.5 C FLAG-CEACAM7

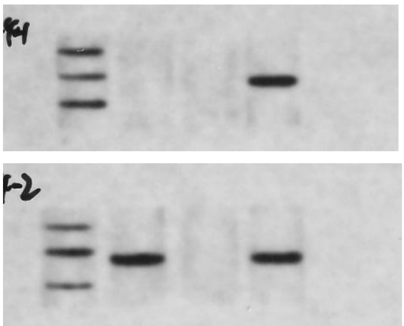

FIG.5 C HA-GCNT3

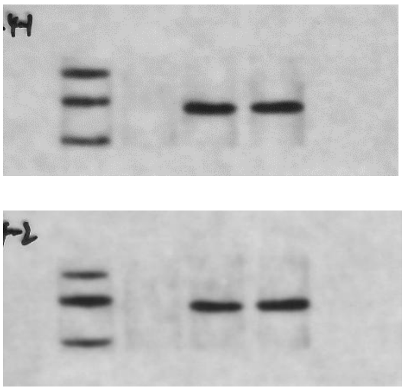

FIG.5 D FLAG-CEACAM7

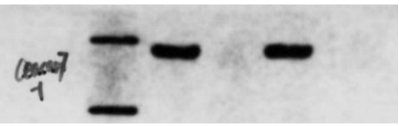

FIG.5 D HA-GCNT3

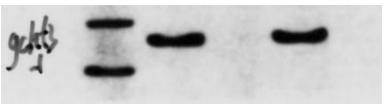

FIG.5 E GCNT3

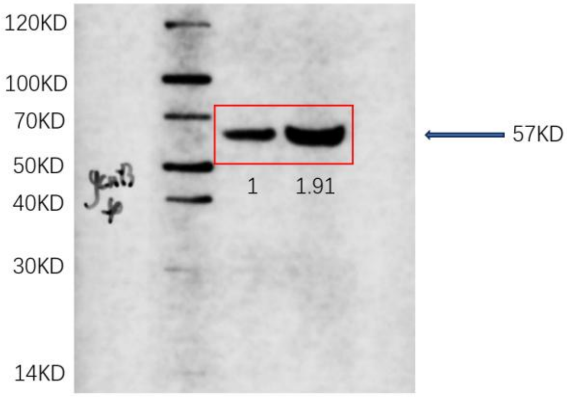

FIG.5 E GAPDH

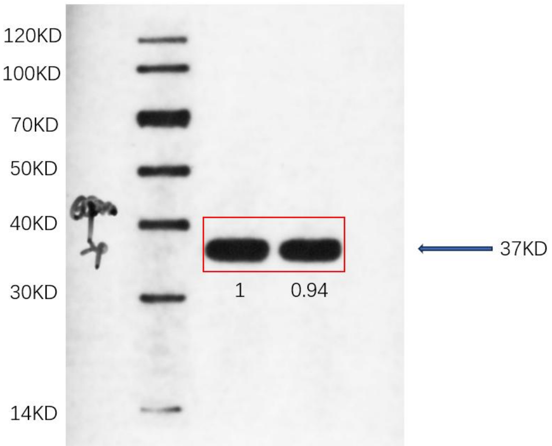

gcnt3	5192	9909
GAPDH	11739	11058
gcnt3/GAPDH	0. 442	0. 896

FIG.5 F GCNT3

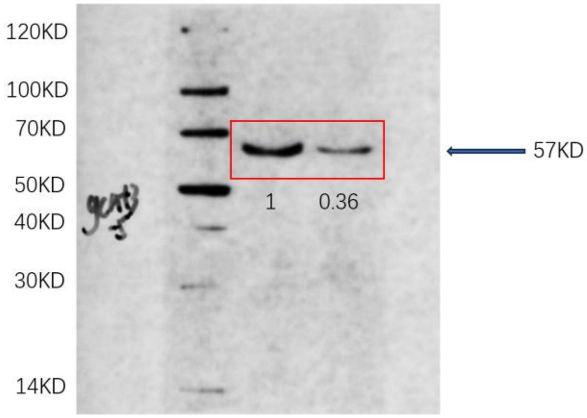

FIG.5 F GAPDH

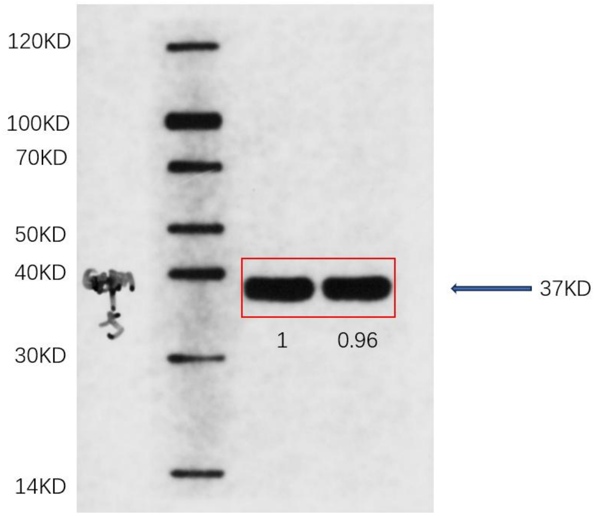

gcnt3	5358	1921
GAPDH	9949	9511
gcnt3/GAPDH	0. 539	0. 202

FIG.6 E E-CAD

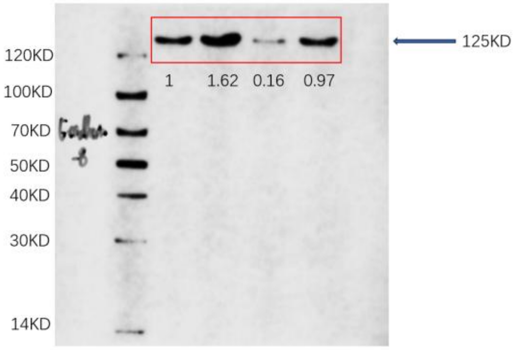

FIG.6 E VIM

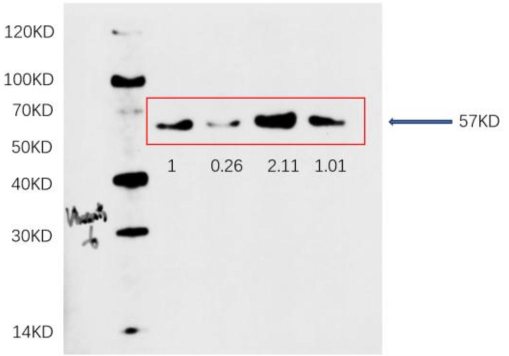

FIG.6 E N-CAD

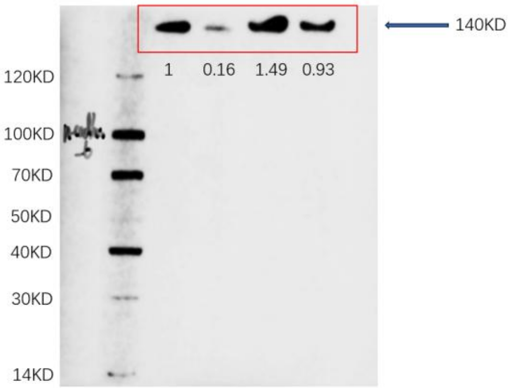

FIG.6 E GAPDH

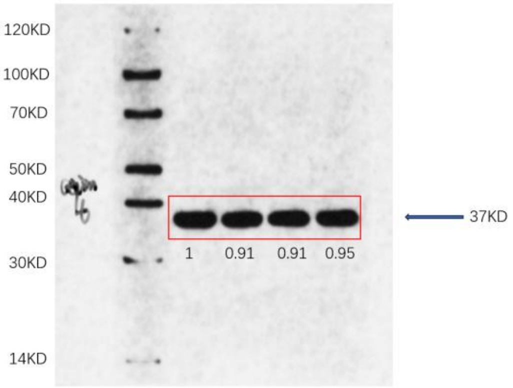

e-cad	4823	7807	749	4663
n-cad	5048	827	7528	4681
vim	3984	1050	8395	4001
GAPDH	10326	9381	9336	9846
e-cad/GAPDH	0. 467	0. 832	0. 080	0. 474
n-cad/GAPDH	0. 489	0. 088	0. 806	0. 475
vim/GAPDH	0. 386	0. 112	0. 899	0. 406

FIG.6 F E-CAD

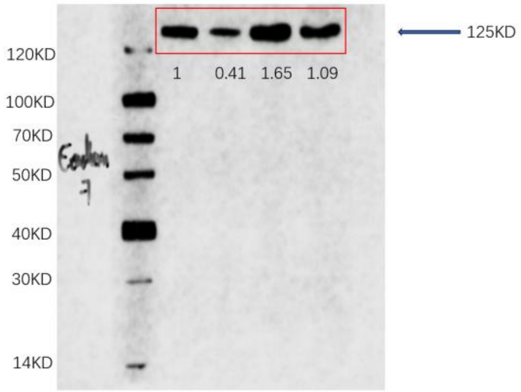

FIG.6 F VIM

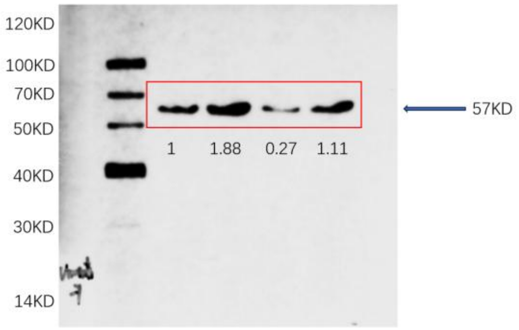

FIG.6 F N-CAD

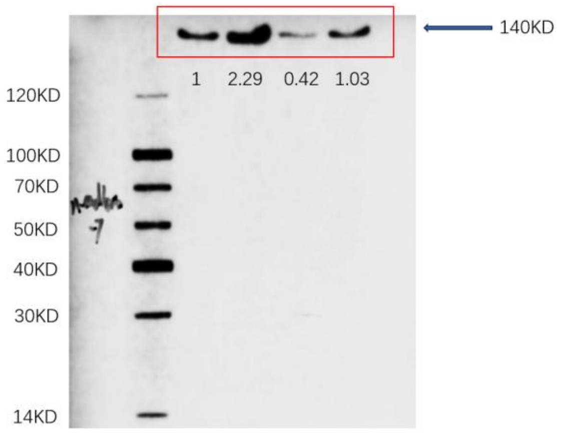

FIG.6 F GAPDH

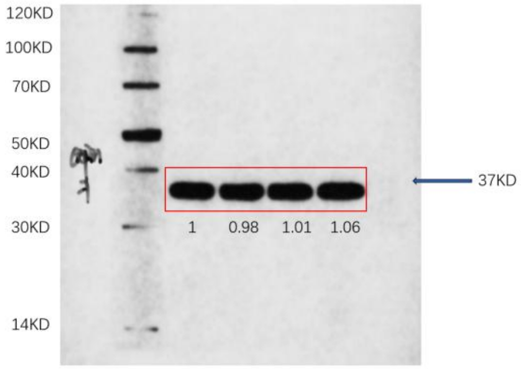

e-cad	4796	1949	7935	5217
n-cad	4279	9789	1785	4416
vim	3186	5990	863	3544
GAPDH	8953	8814	9015	9523
e-cad/GAPDH	0. 536	0. 221	0. 880	0. 548
n-cad/GAPDH	0. 478	1. 111	0. 198	0. 464
vim/GAPDH	0. 356	0. 680	0. 096	0. 372

FIG.7 D GCNT3

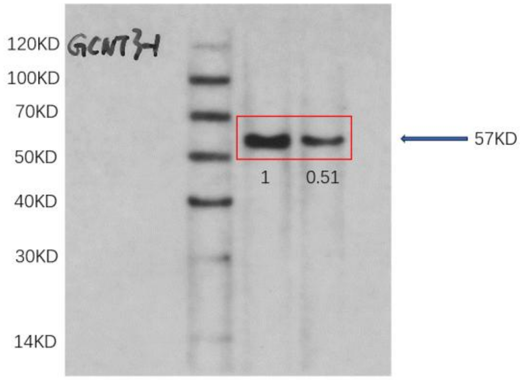

FIG.7 D E-cad

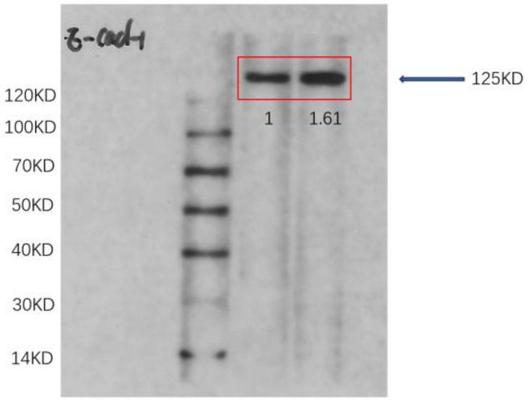

FIG.7 D Vimentin

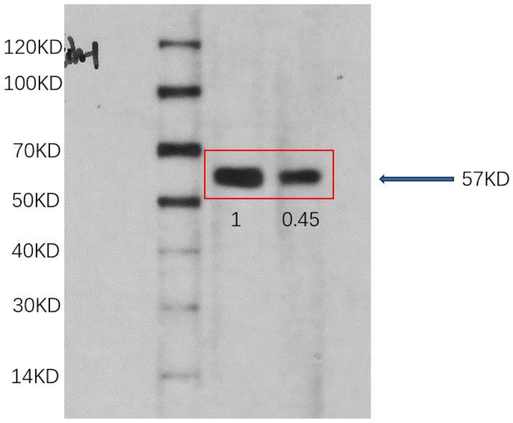

FIG.7 D N-cad

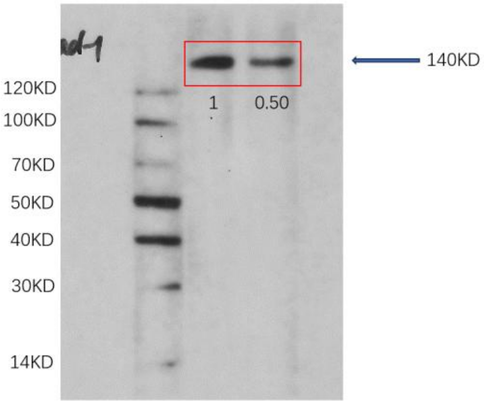

FIG.7 D GAPDH

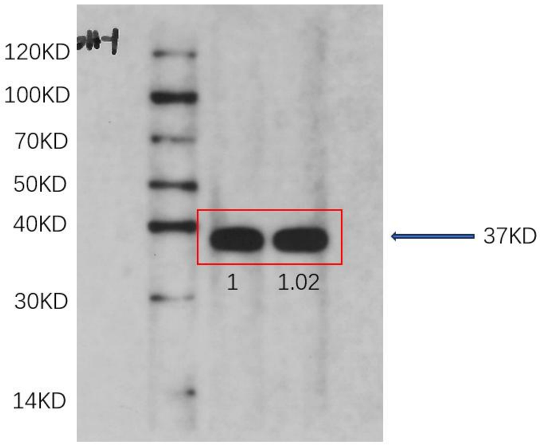

GCNT3	6644	3476
e-cad	5397	8678
n-cad	5258	2605
vim	5387	2405
GAPDH	10710	10932

Supplementary Figure 2 A

CEACAM7

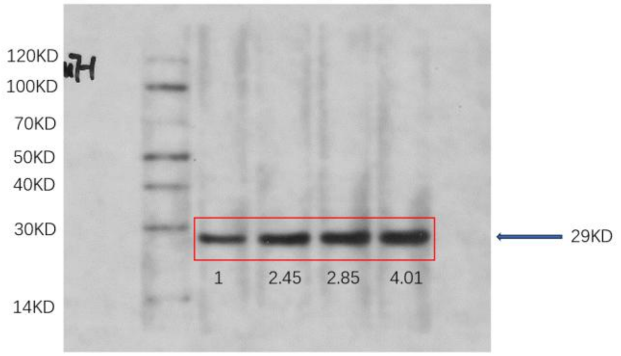

GAPDH

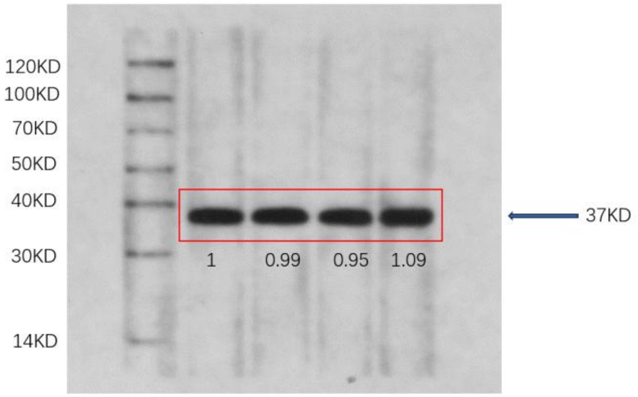

CEACAM7	1179	2887	3355	4802
GAPDH	7930	7905	7529	8652
CEACAM7/GAPDH	0. 149	0. 365	0. 446	0. 555

Supplementary Figure 2 C

CEACAM7

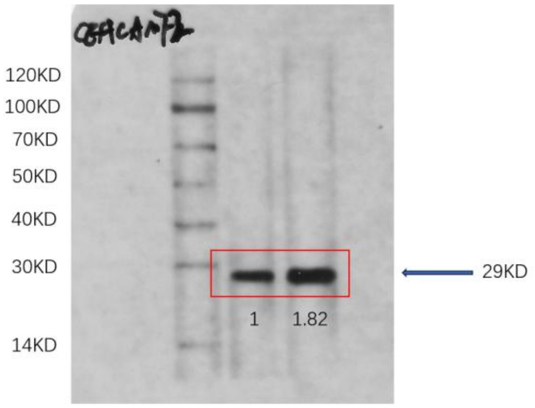

GAPDH

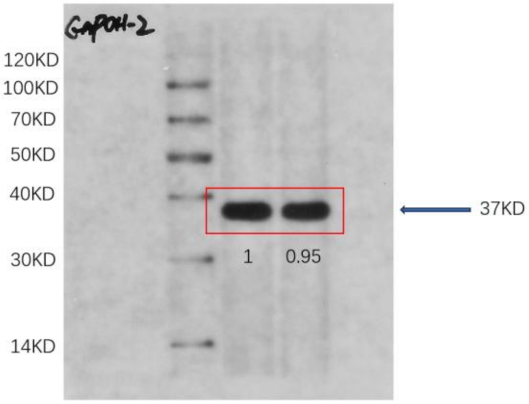

CEACAM7	3586	6512
GAPDH	9595	9114
CEACAM7/GAPDH	0. 374	0. 715

CEACAM7

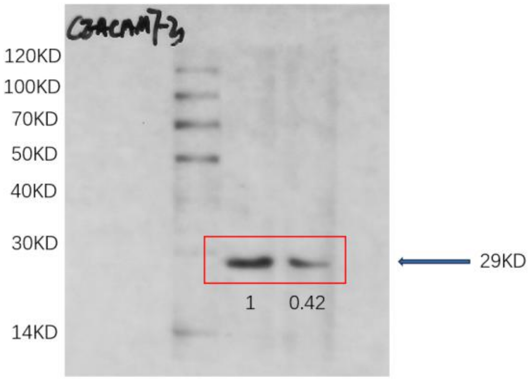

GAPDH

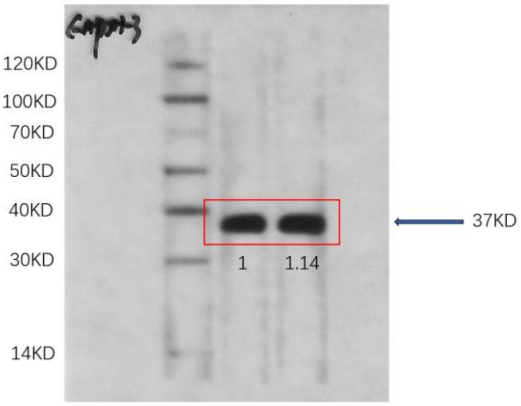

CEACAM7	3021	1269
GAPDH	8111	9226
CEACAM7/GAPDH	0. 372	0. 138

Supplementary Figure 3 A

SLUG

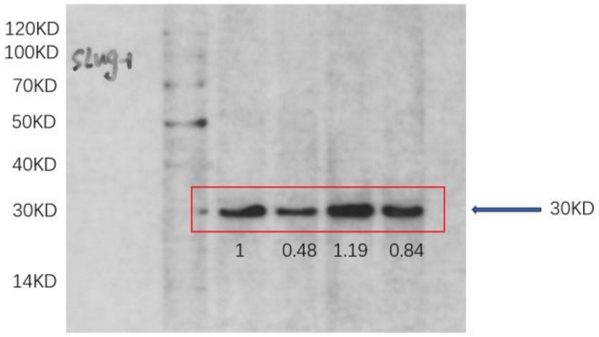

ZEB1

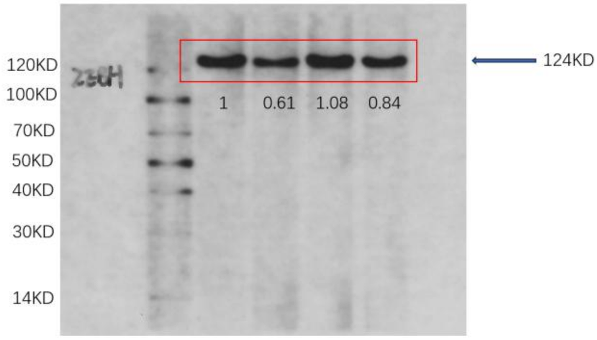

GAPDH

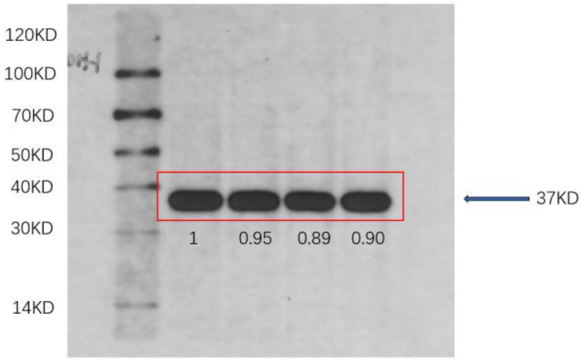

SLUG	6997	3369	8360	5853
ZEB1	7214	4411	7827	6028
GAPDH	11813	11165	10332	10435
SLUG/GAPDH	0. 592	0. 302	0. 809	0. 561
ZEB1/GAPDH	0. 611	0. 395	0. 758	0. 578

Supplementary Figure 3 B

SLUG

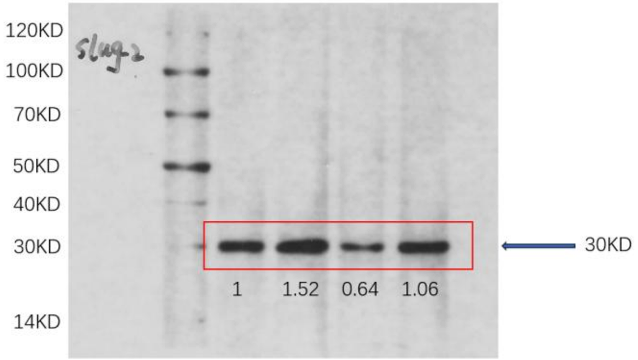

ZEB1

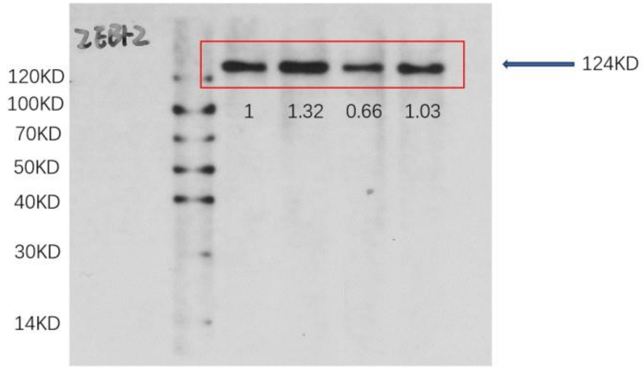

GAPDH

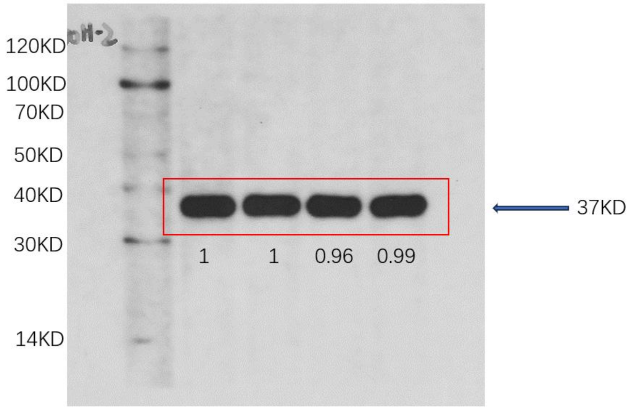

SLUG	6929	10507	4464	7336
ZEB1	7077	9321	4699	7319
GAPDH	11522	11535	11041	11375
SLUG/GAPDH	0.601	0.911	0.404	0.645
ZEB1/GAPDH	0.614	0.808	0.426	0.643
